# Evaluating the prognostic potential of telomerase signature in breast cancer through advanced machine learning model

**DOI:** 10.3389/fimmu.2024.1462953

**Published:** 2024-11-28

**Authors:** Xiao Guo, Yuyan Cao, Xinlin Shi, Jiaying Xing, Chuanbo Feng, Tao Wang

**Affiliations:** ^1^ School of Pharmacy, Beihua University, Jilin, Jilin, China; ^2^ Research Laboratory Center, Guizhou Provincial People’s Hospital, Guiyang, Guizhou, China

**Keywords:** breast cancer, telomerase genes, machine learning, PD-1, gemcitabine

## Abstract

**Background:**

Breast cancer prognosis remains a significant challenge due to the disease's molecular heterogeneity and complexity. Accurate predictive models are critical for improving patient outcomes and tailoring personalized therapies.

**Methods:**

We developed a Machine Learning-assisted Telomerase Signature (MLTS) by integrating multi-omics data from nine independent breast cancer datasets. Using multiple machine learning algorithms, we identified six telomerase-related genes significantly associated with patient survival. The predictive performance of MLTS was evaluated against 66 existing breast cancer prognostic models across diverse cohorts.

**Results:**

The MLTS demonstrated superior predictive accuracy, stability, and reliability compared to other models. Patients with high MLTS scores exhibited increased tumor mutational burden, chromosomal instability, and poor survival outcomes. Single-cell RNA sequencing analysis further revealed higher MLTS scores in aneuploid tumor cells, suggesting a role in cancer progression. Immune profiling indicated distinct tumor microenvironment characteristics associated with MLTS scores, potentially guiding therapeutic decisions.

**Conclusions:**

Our findings highlight the utility of MLTS as a robust prognostic biomarker for breast cancer. The ability of MLTS to predict patient outcomes and its association with key genomic and cellular features underscore its potential as a target for personalized therapy. Future research may focus on integrating MLTS with additional molecular signatures to enhance its clinical application in precision oncology.

## Introduction

Breast cancer, historically labeled as the “invisible killer” in middle-aged and older women, has alarmingly started to proliferate among younger demographics at an unprecedented rate. Recent findings from the International Agency for Research on Cancer reveal a striking 24.2% incidence rate of breast cancer, underscoring a critical, escalating public health issue that continues to rise annually (1).

While various prognostic models have been developed, such as those by Sui et al. using immune cell infiltration scores (2), and by Elke M. et al. integrating genetic polymorphisms (3), the overall accuracy and effectiveness of these models in predicting patient outcomes remain suboptimal. This underscores a compelling need for innovative approaches that enhance predictive accuracies which could significantly alter treatment paradigms. Telomerase can prevent telomeres from shortening with the increase in the frequency of cell division, thus achieving the infinite cell replication of cells (4). This activity is inhibited in normal cells. However, some studies have shown that the expression rate of telomerase in breast cancer, oral squamous cell carcinoma, gastric cancer, lung cancer, liver cancer, and ovarian cancer is significantly higher than that in benign lesions and normal tissues (5), suggesting that telomerase helps tumor cells evade the limitations of cell aging and death to promote their unlimited proliferation, so telomerase has become one of the hotspots in cancer research.

This study introduces a novel predictive model based on telomerase gene expressions, utilizing advanced machine learning techniques to refine prognosis evaluations further. By integrating comprehensive bioinformatics data, this research not only aims to bridge the gap identified in traditional models but also enhances the understanding of telomerase’s role in cancer progression—a pivotal step in tailoring patient-specific therapeutic strategies.

## Methods

### Data Acquisition

We compiled data from 12 independent breast cancer cohorts sourced from three major databases: The Cancer Genome Atlas (TCGA), the Gene Expression Omnibus (GEO), and MetaGxData. Our analysis focused on samples that included complete survival data to ensure the reliability and comprehensiveness of the prognostic analysis. Genes for telomere maintenance were collected from the TelNet database (6).

### Machine learning-assisted telomerase signature for breast cancer

Following the methodology of Liu et al. (7), we integrated ten computational tools to develop a unique telomerase signature for breast cancer: Random Forest (RF), Least Absolute Shrinkage and Selection Operator (LASSO), Gradient Boosting Machine (GBM), Survival Support Vector Machine (Survival-SVM), Supervised Principal Component (SuperPC), Ridge Regression, Partial Least Squares Cox Regression (plsRcox), CoxBoost, Stepwise Cox Regression, and Elastic Net (Enet). Random Forest was used to identify the most predictive genes by constructing multiple decision trees, while LASSO was utilized for feature selection by shrinking the regression coefficients of less significant features to zero. GBM was employed as a boosting algorithm to build a sequence of weak learners to progressively reduce the residual error in predictions, and Survival-SVM modeled survival data by maximizing the margin between survival time points of different classes. Hyperparameters for each algorithm were optimized using grid search with ten-fold cross-validation; for example, the number of trees in RF was set to 1000, while the regularization parameter in LASSO was tuned to minimize prediction error. RF, LASSO, CoxBoost, and Stepwise Cox were particularly critical for dimensionality reduction and variable selection, combined into 108 distinct configurations to generate a predictive signature. We evaluated all cohorts, including TCGA and five GEO datasets, using the average Concordance Index (C-index) to identify the most reliable prognostic model, establishing a redox-specific signature to predict outcomes in breast cancer.

### Genomic alteration analysis in MLTS groups

We investigated genetic variations between two MLTS groups using the TCGA-BRCA database, analyzing both genetic mutation levels and Copy Number Alterations (CNA). The Tumor Mutation Burden (TMB) for high- and low-MLTS breast cancer patients was calculated from raw mutation data, and the most frequently mutated genes (mutation rate > 5%) were visualized using maftools. Additionally, patient-specific mutational signatures were identified with the deconstructSigs tool (8). Mutational analysis was conducted to visualize the mutation spectrum across different MLTS subgroups, with TMB calculated by counting the number of mutations per megabase and results presented in box plots comparing the groups. Copy number alterations were detected using GISTIC2.0 to identify significant amplification and deletion events across the genome, which were visualized using heatmaps to illustrate the distribution of these alterations in the MLTS subgroups. We highlighted four prominent mutational signatures (SBS2, SBS13, SB7b, SBS7d) showing higher mutation frequencies in the dataset, and noted the five most common regions of amplification and deletion, particularly in genes located at 5p15.33 and 9p23.

### Single-cell RNA sequencing data processing

To analyze single-cell RNA sequencing data, we processed the GSE161529 dataset using Seurat (v4.0) (9). Initially, genes without expression were removed, focusing only on those with nonzero expression levels. We normalized the expression matrix using Seurat’s “SCTransform” function and reduced dataset dimensionality through PCA and UMAP techniques. Distinct cellular groupings were identified via “FindNeighbors” and “FindClusters” functions. The DoubletFinder package was employed to eliminate potential doublets, ensuring dataset integrity (10). Cells not meeting quality standards—such as those with over 15% mitochondrial genes or fewer than 500 genes—were excluded. Ultimately, 64,308 cells met our rigorous quality control criteria and were further categorized by manually annotating cell types based on established marker genes.

### Adapting SCENIC for gene regulatory network inference

The Single-Cell rEgulatory Network Inference and Clustering (SCENIC) pipeline is a computational method used to reconstruct gene regulatory networks from single-cell RNA sequencing (scRNA-seq) data (11). SCENIC identifies co-expressed gene modules, determines direct regulatory targets based on transcription factor motif enrichment, and defines regulons (transcription factors and their target genes): 1. We first identified co-expression modules using the GENIE3 algorithm, which ranks genes based on their importance as potential transcription factor targets. This approach allows us to detect modules of genes that are likely regulated by the same transcription factors. 2. For each co-expression module, SCENIC applies motif enrichment analysis using RcisTarget to identify direct targets of transcription factors. Only genes with enriched binding motifs are retained as part of a regulon, ensuring that the predicted gene-target interactions are supported by sequence-specific evidence. 3. We used the AUCell algorithm to calculate the regulatory activity score (RAS) for each cell, based on the expression of genes within each regulon. This scoring system quantifies the activity of each regulon in individual cells, enabling the identification of cell states driven by specific transcription factors (12). 4. Using dimensionality reduction techniques (e.g., UMAP), we visualized the distribution of cells based on regulon activity scores. Cells with similar transcriptional regulatory profiles were grouped into clusters, highlighting distinct cell states within the breast cancer microenvironment.

The SCENIC pipeline enabled us to identify key transcription factors and their target genes that drive specific cell states in breast cancer. This approach provided insights into the gene regulatory networks underlying tumor progression and immune response, revealing potential therapeutic targets for modulating these pathways.

### Regulon clustering in regulatory crosstalk analysis

Our study utilizes a sophisticated computational approach to map the regulatory crosstalk among TFs and their target genes, emphasizing TF clustering. The method begins by filtering TF-target interaction data to focus on significant pairs (significance threshold > 1), prioritizing the most relevant regulatory interactions. We identify key regulatory TFs, termed hub genes, by quantifying their target gene regulation. An undirected graph model represents these interactions, refined spatially by a force-directed algorithm to clearly depict the network architecture, and highlight TF-target interplay. Further structural insights are gained through the Leiden algorithm, which detects community structures and groups TFs into clusters based on their regulatory links, enhancing our analysis of the regulatory landscape.

### Cell-cell communication analysis

We utilized the “CellChat” R package to analyze cell-cell communication (13), creating CellChat objects from UMI count matrices and employing the “CellChatDB.human” database for ligand-receptor interactions. Analysis was performed using default settings, with objects merged via the “mergeCellChat” function. Interaction differences in number and intensity across cell types were visualized using “netVisual_diffInteraction.” Changes in signaling pathways were assessed with “rankNet,” and gene expression distributions were depicted using “netVisual_bubble” and “netVisual_aggregate.”

Additionally, the NicheNet package provided insights into ligand activity and regulated expression of downstream targets (14), enhancing our understanding of signaling dynamics and communication pathways in the cellular microenvironment.

### Evaluation of TME disparities and immunotherapy response

To assess immune cell infiltration in the tumor microenvironment (TME), we utilized several algorithms: MCPcounter (15), EPIC (16), xCell (17), CIBERSORT (18), quanTIseq (19), and TIMER (20). These analyses helped categorize patients by their MLTS scores and provided a comprehensive view of the immune landscape. Additionally, we evaluated the ESTIMATE and TIDE indices to gain insights into immunotherapy potentials and prognostic implications for breast cancer (21, 22). Immune checkpoints were quantified to predict patient responsiveness to immune checkpoint inhibitor (ICI) therapy, supporting personalized medicine and improved treatment strategies.

### Therapeutic target and drug identification for High MLTS patients

To identify potential therapies for high MLTS patients, we initially filtered duplicate compounds from the Drug Repurposing Hub, narrowing down to a list of 6,125 unique compounds (https://clue.io/repurposing). We used Spearman correlation analysis to select genes linked with breast cancer outcomes, focusing on those with a correlation coefficient greater than 0.15 (P < 0.05) and those indicating poor prognosis with coefficients below -0.30 (P < 0.05). We also evaluated gene significance using CERES scores from the Cancer Cell Line Encyclopedia (CCLE) related to brain cell risk scores (23).

Further, we assessed drug responsiveness using data from the Cancer Therapeutics Response Portal (CTRP) and the PRISM project, which include drug screening across cancer cell lines. The predictive accuracy of drug responses was enhanced using the pRRophetic package’s ridge regression model, validated by 10-fold cross-validation (24).

Additionally, we explored potential drugs using Connectivity Map (CMap) analysis by comparing gene expression profiles and identifying compounds inversely related to CMap scores, suggesting higher therapeutic potential against breast cancer.

### Patient stratification in breast cancer research

For gene expression analysis in breast cancer samples, RNA was extracted using TRIzol reagent (Invitrogen, Carlsbad, CA, USA), and cDNA was synthesized using GoScript reverse transcriptase and Master Mix (Promega), following manufacturer’s protocols. Quantitative expression was measured via qRT-PCR on the CFX96 Touch Real-Time PCR Detection System (BioRad, Hercules, CA, USA) using the 2^-ΔΔCq^ method with GAPDH as a normalization control. Patients were then categorized based on gene expression levels calculated from a formula derived from the MLTS, which helped identify varying risk profiles and assisted in developing customized treatment approaches.

### Immunohistochemistry analysis

We collected breast cancer tissue samples from 30 patients at Guizhou Provincial People’s Hospital and performed Hematoxylin and Eosin (HE) staining according to established protocols, with diagnoses confirmed by two independent pathologists. For the immunohistochemistry (IHC) analysis on paraffin-embedded samples, we followed the procedures and scoring systems described in our previous studies (25, 26). Protein expression levels were assessed independently by the same pathologists, ensuring methodological consistency with our earlier research (26).

## Results

### Construction of telomerase gene signature based on machine learning

**Figure 1:**
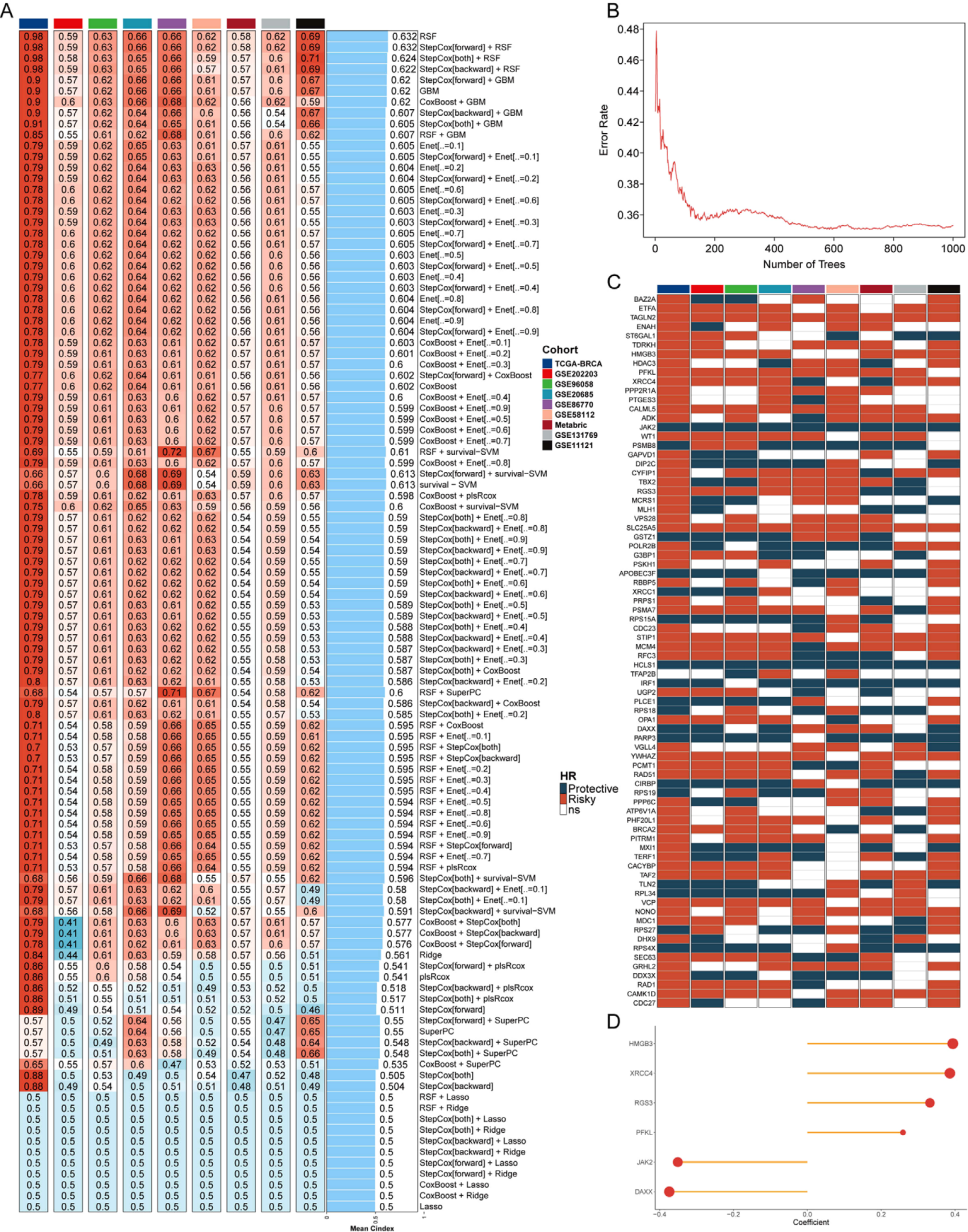
Machine learning and development of telomerase gene model. **(A)** C index values of 9 datasets in different machine learning combinations. **(B)** The error rates of RSF algorithm running 1000 times. **(C)** Prognostic outcomes of different important genes in 9 datasets. **(D)** Coefficients of 6 genes for model construction.

To investigate the relationship between breast cancer and telomerase genes in depth, the information related to telomerase genes was collected from TelNet database to ensure the comprehensiveness and accuracy of the data. An in-depth analysis was conducted on the 9 independent datasets, and ten-fold cross-validation and 108 algorithm combinations were applied to construct a machine learning-assisted telomerase signature (MLTS). Subsequently, C-index values of the algorithms were calculated and compared to select the best algorithm (**Figure 1A**). The heatmap highlights the performance of each algorithm across the datasets, with red colors indicating higher C-index values, which signify better predictive performance. The RSF was identified as the most robust model, displaying the highest C-index values.

The telomerase genes may regulate the breast cancer process, and the individual assessment on the important genes contained in the point with the lowest error rate was conducted by RSF algorithm (**Figure 1B**). The error rate for the RSF algorithm is plotted against the number of trees, showing that as the number of trees increases, the error rate significantly decreases and stabilizes around 400 trees. This suggests that the RSF model reached optimal performance at this level, making it a reliable choice for gene selection. The prognostic value of these key telomerase genes was explored by univariate COX regression analysis, in which some typical oncogenes, such as HMGB3, XRCC4, RGS3, and PFKL, were found to be positively correlated with the poor prognosis, and some typical tumor suppressor genes, such as JAK2 and DAXX, were also found to be negatively correlated with the poor prognosis in breast cancer patients (**Figure 1C**). The six screened genes were used to construct a MLTS (**Figure 1D**), and the survival of the binary classification model was verified by calculating MLTS scores in 9 cohorts. The results showed that the binary classification model could effectively distinguish between the high- and low-MLTS patients, indicating that MLTS can provide some important reference information for predicting the survival of breast cancer patient (**Supplementary Figure S1**).

### Evaluation of MLTS using 66 published breast cancer models

The independence between MLTS and other clinical indicators was compared by univariate and multivariate COX analysis (**Supplementary Figure S2A**), and MLTS, stage, and age were ultimately used to establish a nomogram for evaluating the patient’s OS at 1, 3, and 5 years (**Supplementary Figure S2B**). The calibration curve analysis showed that the OS predicted by the nomogram was highly consistent with the observed OS (**Supplementary Figure S2C**). In addition, Hosmer-Leme analysis found no significant difference between the MLTS nomogram curve and the ideal curve (**Supplementary Figure S2D**). The DCA results showed that the net benefit of the MLTS curve was much higher than that of the other two curves (**Supplementary Figure S2E**). At last, the ROC curve of MLTS was relatively higher than other traditional factors (**Supplementary Figure S2F**).

**Figure 2:**
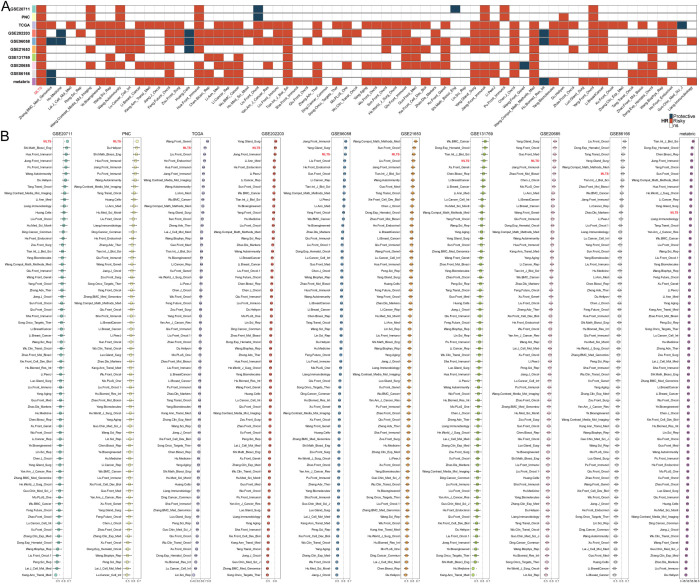
Comparison between the MLTS and 66 published models. **(A)** Univariate Cox regression analysis of MLTS and 66 published signatures. **(B)** C-indices of MLTS and 66 published signatures in 10 datasets.

To comprehensively evaluate the predictive ability of MLTS, 66 breast cancer prediction models developed by different research teams were collected, and the stability and accuracy of these 67 models (including MLTS) were evaluated in 10 independent datasets. Heatmap summarizes the stability of each model across the datasets, with MLTS consistently performing as the most stable model (**Figure 2A**). In addition, average C-index values calculated by the different model in 10 datasets a comparison were compared, and the comparison results indicated that MLTS had a high accuracy and stability (**Figure 2B**). The plot illustrates that MLTS achieved the highest average C-index across the datasets, demonstrating its superior predictive accuracy compared to other models.

### Gene mutation landscape of MLTS

**Figure 3:**
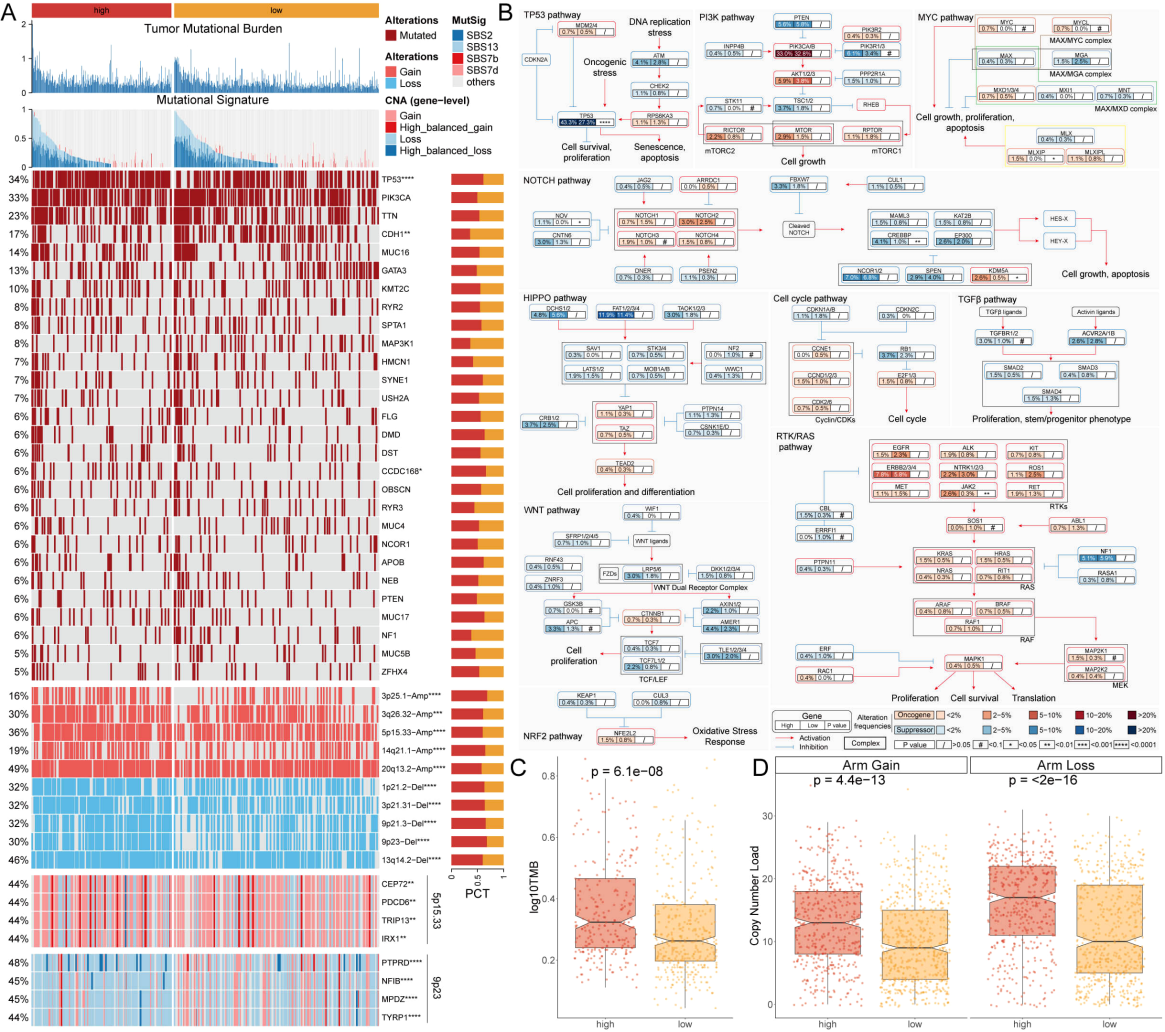
Genomic and pathway alterations associated with MLTS. **(A)** Displays the TMB and various mutational signatures across the genome. A detailed heatmap below illustrates the frequency of specific genomic alterations, such as gains and losses, with adjacent bar graphs quantifying these alterations for selected genes. **(B)** Mutation map for genes implicated in ten canonical cancer pathways, revealing the complexity of oncogenic signaling and the interactions among pathways in tumorigenesis. **(C, D)** Box plots compare TMB **(C)** and copy number variations D). *p < 0.05, **p < 0.01, ***p < 0.001, and ****p < 0.0001.

Due to the important role of gene mutations in the occurrence and development of cancer, differences in the genomic level between MLTS subgroup were deeply explored by using a multi-omics integrated analysis method. Firstly, the tumor mutation burden (TMB) of patients in the two groups was compared (**Figures 3A, C**). The top panel of **Figure 3A** illustrates the distribution of TMB between the high- and low-MLTS groups, showing that the high-MLTS patients tend to have a significantly higher TMB, as confirmed by the box plot in **Figure 3C**. The study focused on the analysis of genes with higher point mutation rates and variations in their chromosome copy numbers, and it was found that the mutation and variation in these genes were more frequent in the high-MLTS patients (**Figure 3A**). Each row in heatmap represents a gene with a high mutation frequency, with darker shades indicating higher mutation rates. High-MLTS patients show a more pronounced mutation profile, especially in genes such as TP53, highlighting the potential link between these mutations and poor prognosis.

Finally, combined with the data from 10 carcinogenic signaling pathways, the mutation rates of important genes were observed. The results showed that the mutation rate of some classic tumor suppressor genes was significantly higher in the high-MLTS group. For example, the mutation rate of TP53, a typical tumor suppressor gene, was 43.3% in the high-MLTS group, and only 27.3% in the low-MLTS group. (**Figures 3A, B**). The maps the involvement of these mutated genes in critical signaling pathways, such as the TP53 and PI3K pathways, where nodes are color-coded to indicate the mutation frequency, further linking the pathway alterations to the aggressive tumor behavior observed in high-MLTS patients.

Then, the copy number alteration of patients in MLTS were further explored, and it was found that the amplification and deletion of chromosomes at the chromosome arm level were more pronounced in the high-MLTS group (**Figures 3A, D**), indicating that the poor prognosis of high-MLTS patients may be related to the significant increase of multiple oncogenes such as 5p15.33 and the deletion of multiple tumor suppressor genes such as 9p23 (**Figure 3A**).

### Interpretation of biological mechanisms of MLTS at the single-cell level

**Figure 4:**
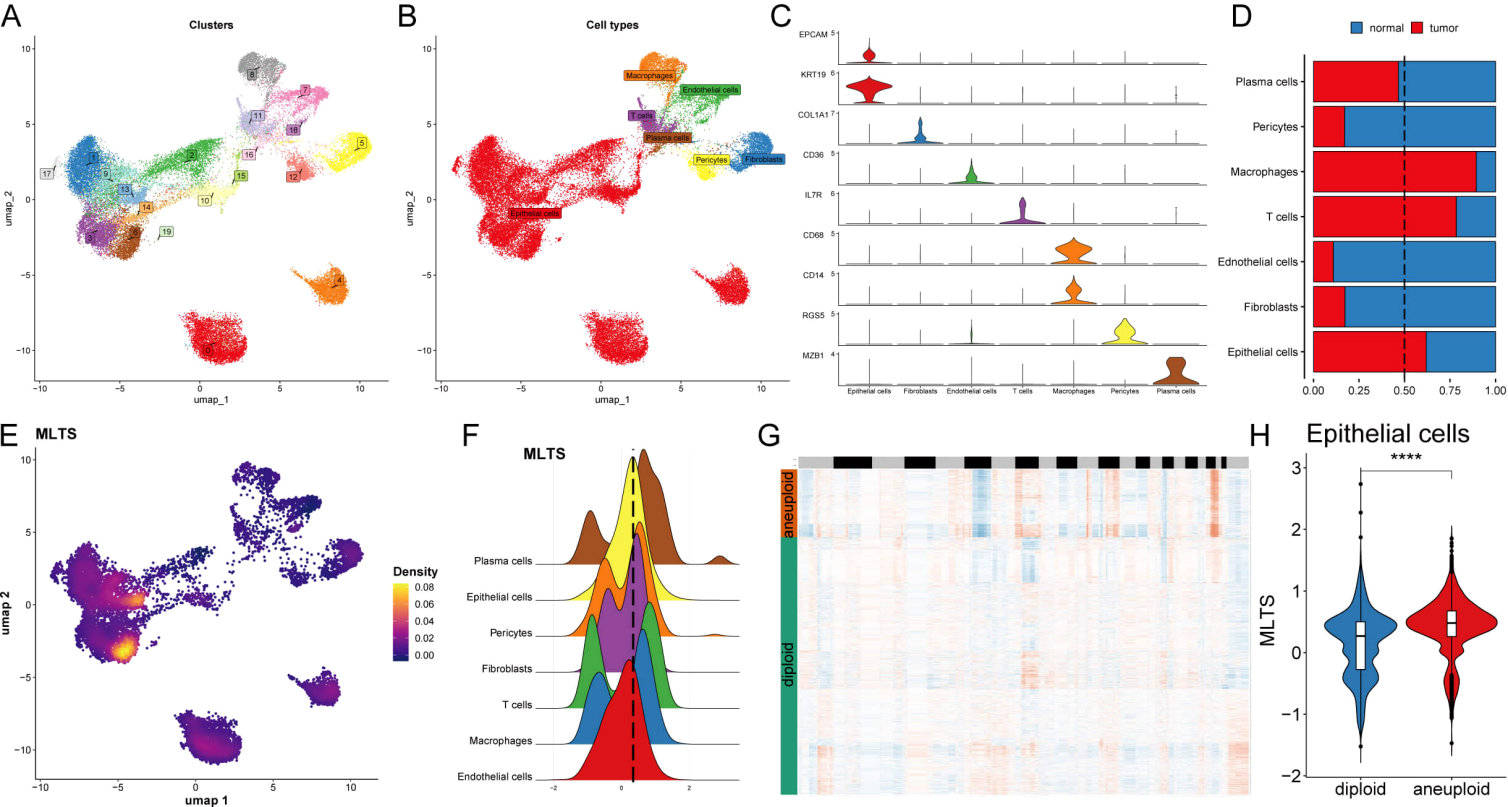
Single-cell analysis of cellular heterogeneity and MLTS level. **(A)** A UMAP visualization identifies various clusters, each color representing a distinct cluster. **(B)** UMAP plot is color-coded to delineate different cell types within the sample. **(C)** Violin plots illustrate the expression levels of specific marker genes across different cell types, providing insights into the relative abundance or activity of these cells. **(D)** A bar chart shows the proportion of each cell type in normal versus tumor samples, comparing cellular composition. **(E)** A UMAP density plot visualizes MLTS value across different cell populations, with warmer colors indicating higher expression levels. **(F)** A violin plot reveals the distribution of MLTS value across various cell types. **(G)** A heatmap by CopyKAT displays inferred copy number variations for a range of genes (rows) against individual cells (columns), where color intensity indicates the extent of aneuploidy or copy number alterations. **(H)** A violin plot contrasts the MLTS score between diploid and aneuploid cells within the epithelial cell population, highlighting significant genomic differences. ****p < 0.0001.

The single-cell distribution of 8 breast cancer patients was analyzed, maps of the single-cell distribution in four normal tissues and four tumor tissues are included (**Supplementary Figures S3A, B**). Single-cell distribution maps of these 8 breast cancer patients were analyzed after removing the batch effect, and 20 clusters and seven cell types were identified. (**Figures 4A, B**). The UMAP plot shows distinct clusters representing the 20 different cell populations and specific cell types to these clusters, each color-coded to highlight its identity, indicating the diversity of cell populations within the tumor microenvironment. The overall number of these 7 types of cells and their proportion in the bodies of these 8 tumor patients was statistically analyzed (**Supplementary Figures S3C, D**). The representative markers in these 7 types of cells and their actual distribution within the cells were observed (**Figure 4C**; **Supplementary Figure S3E**). Violin plots of key marker genes for each cell type, illustrating their expression profiles across the identified populations, which helps confirm the identity of these cells in both normal and tumor tissues. Moreover, the distribution of these 7 types of cells in the tumor tissue and normal tissue was summarized. We observed that epithelial cells, macrophages, and T cells accounted for a larger proportion in the tumor tissue in comparison with normal tissue (**Figure 4D**), indicating that tumor tissues have a higher prevalence of these immune and epithelial cells compared to normal tissues, suggesting their involvement in the tumor’s immune landscape and progression.

Next, MLTS was incorporated into the single-cell analysis for scoring (**Figure 4E**). The cells were divided into two groups based on peak MTLS scores of the epithelial cells (**Figure 4F**). Differential gene expression analysis and functional clustering were performed on these 7 cell types to elucidate the potential functional pathways of MTLS (**Supplementary Figures S3F, G**). Further observation on the copy-number alteration by copyKat algorithm was used to distinguish between normal cells and tumor cells (**Figure 4G**), and finally a higher MTLS score in tumor-aneuploid than in tumor-diploid, implying the significance of MTLS in breast cancer progression (**Figure 4H**).

### Exploring specific regulatory factors that drive MLTS and cell recognition

**Figure 5:**
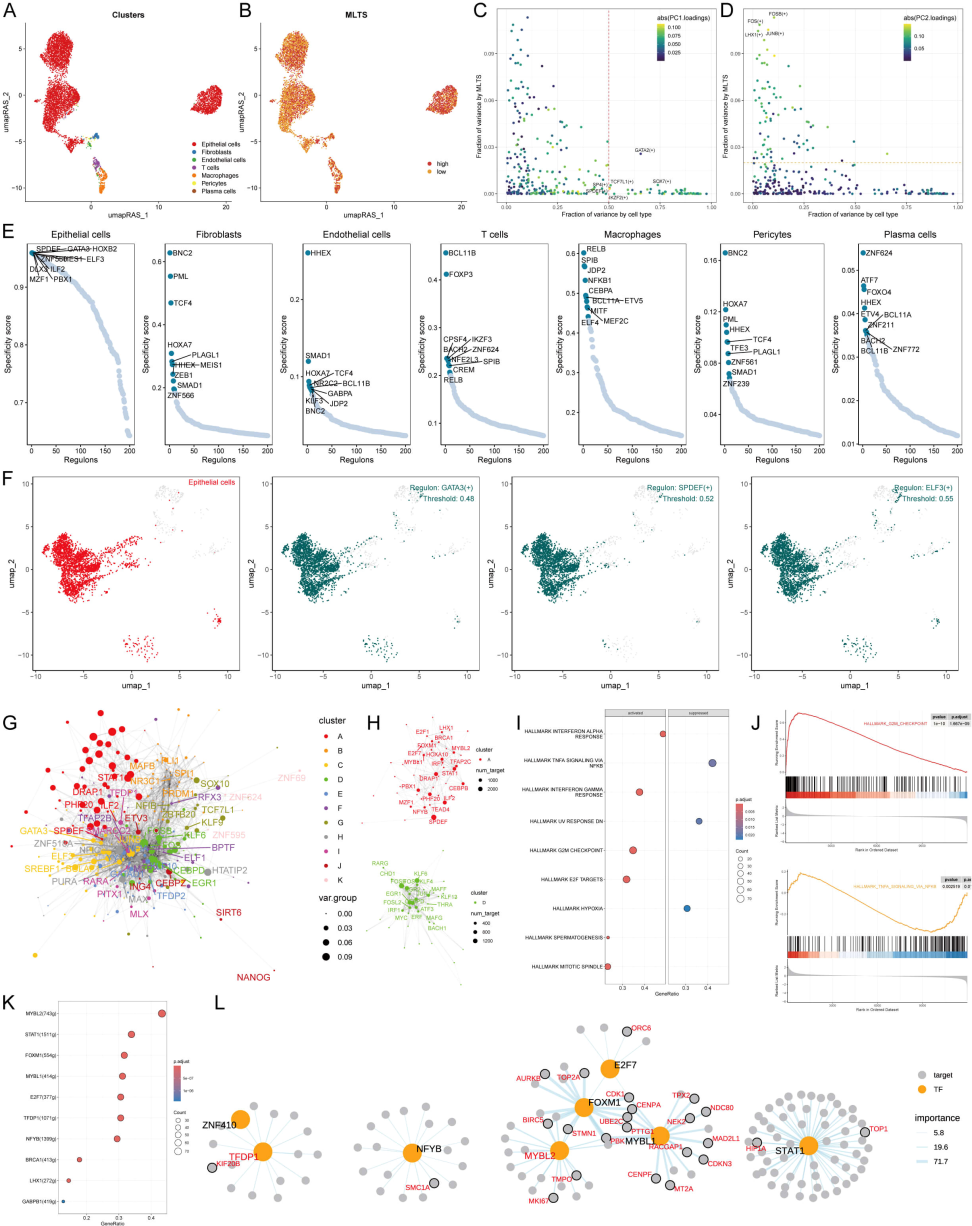
MLTS-specific regulon activity analysis. **(A)** A umapRAS plot illustrates distinct clusters within a cell population, with each color representing a unique cluster, suggesting differences in cell subtypes or states. **(B)** This umapRAS plot further depicts MLTS levels across the cell population, with varying color intensities reflecting the magnitude of scores. **(C)** A variance analysis plot highlights the impact of cell types and MLTS on transcription factor activity, using color mapping to PC1 to emphasize the primary variance influenced by these factors. **(D)** Another variance analysis plot, color-mapped to PC2, explores additional dimensions of variability. **(E)** Displays a ranking for regulons in each cell type based on Regulon Specificity Score (RSS). **(F)** UMAP plots specifically focus on epithelial cells, each showing areas where a particular regulon is active. **(G)** A network graph, constructed via the Leiden algorithm, mapped the complex interactions among them. **(H)** This graph concentrates on modules A and D, which significantly contribute to MLTS, elucidating their role in regulon dynamics. **(I)** GSEA identifies pathway variations linked to MLTS in epithelial cells. **(J)** Representative pathways activated or inhibited in the context of high MLTS, indicating specific pathway engagement within epithelial cells. **(K)** Lists transcription factors involved in G2M checkpoint, highlighting their roles in MLTS progression. **(L)** A detailed regulatory network diagram illustrates the interactions among transcription factors involved in G2M checkpoint, delineating their interconnected roles in driving MLTS progression.

To comprehensively construct a gene regulatory network, the SCENIC pipeline was applied to analyze single-cell RNA seq data with cis-regulatory sequence information (**Figures 5A, B**). The PCA and variance analyses were performed to explore the specific regulons based on the MLTS and cell identity. Results showed that PC1 accounted for cell type-specific TFs, while PC2 was correlated with MLTS-specific TFs (**Figures 5C, D**; **Supplementary Figures S4A, B**). The activity and expression levels of NFICH and START1 in 7 different cells were analyzed, and the results showed that START1 was activated in all high-MLTS cells, but the expression levels did not show significant changes, while NFIC was exactly the opposite (**Supplementary Figures S4C, D**).

Based on the Jensen Shannon divergence, the regulator specific score (RSS) was defined, and the regulatory factors with higher RSS scores were selected from these 7 types of cells for matrix analysis. The results showed that GATA3, SPDEF; ELF3 were the most relevant specific regulators to epithelial cells, the UMAP plot provided an additional support for the highly specific activity of these regulators to epithelial cells, and the most relevant specific regulators in the other 6 types of cells were also analyzed (**Figures 5E, F**; **Supplementary Figure S4E**).

Next, the RAS score similarity of each transcription factor was compared based on Leiden algorithm, and 11 transcription factor clusters were obtained by cluster analysis, of which A and D transcription factor sets had a higher contribution rate to the development of MLTS compared to the other 9 groups (**Figures 5G, H**; **Supplementary Figure S4F**). We next focused on the exact TFs that drive epithelial cells transcriptomic changes by MLTS. Multiple mutation pathways were identified by GSEA analysis (**Figures 5I, J**). Transcription factors that contributed to these two representative pathways were identified by further analysis (**Figures 5K**; **Supplementary Figure S4G**). **Figures 5L**; **Supplementary Figure S4H** show the regulatory network diagrams of these transcription factors, such as KYNU which is associated with this activation pathway, and the transcription factors involved in the progression of MLTS was confirmed.

### Intercellular communication between MLTS groups

**Figure 6:**
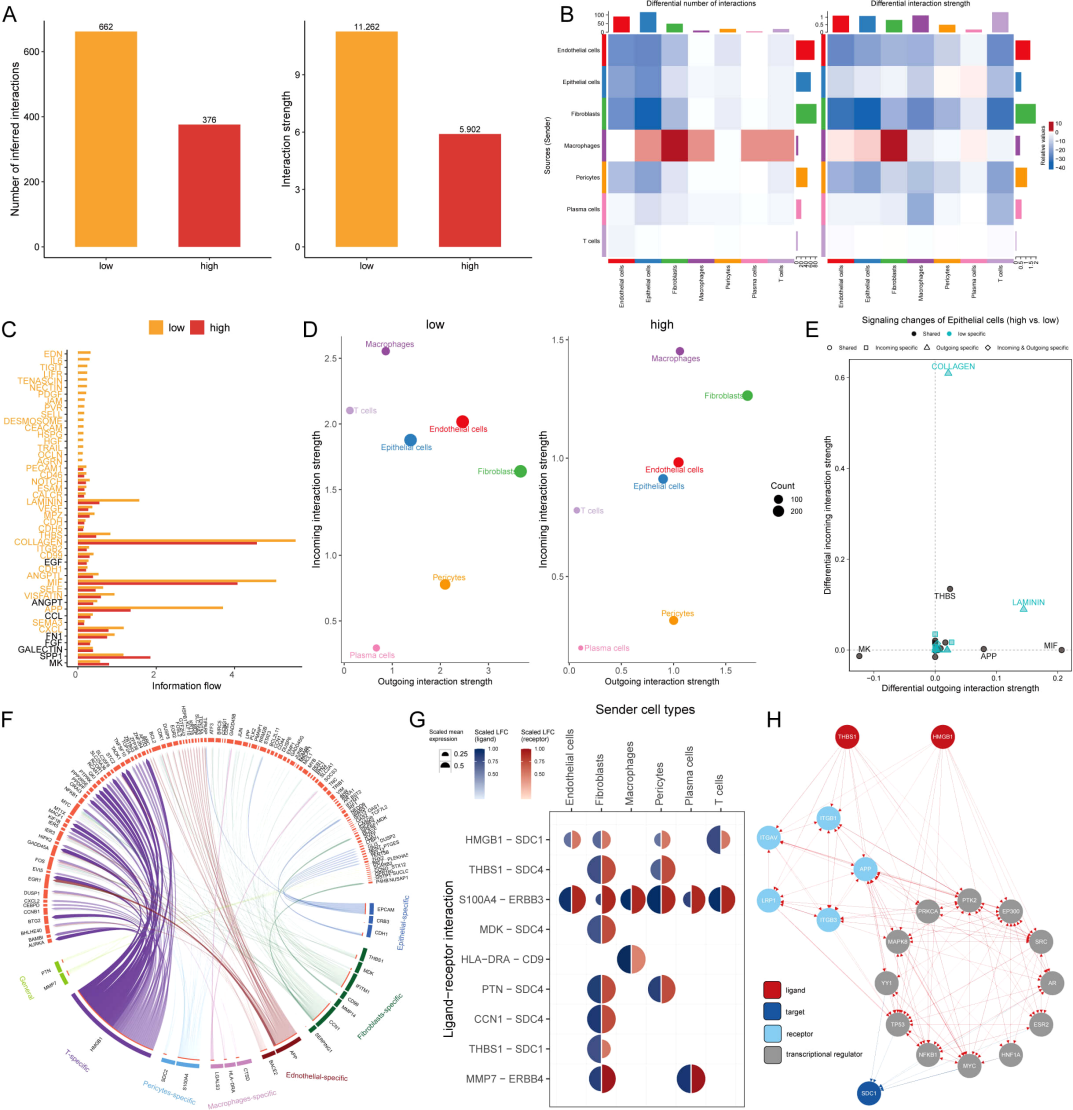
Dissecting cell-cell interactions and signaling pathway dynamics in MLTS-modulated breast cancer progression. **(A)** Bar graphs depict a decrease in cell-cell interactions within the high MLTS group compared to the low. **(B)** Network diagrams illustrate varied interaction strengths among cell types in high MLTS groups. **(C)** A bar chart displays the information flow across 38 signaling pathways, showing that most are more active in the low MLTS group, particularly collagen and MIF pathways crucial for tumor microenvironment structuring. **(D)** Scatter plots compare outgoing and incoming interaction strengths between cell types in low and high MLTS. **(E)** Pathway specificity in epithelial cells within high MLTS includes notably specific pathways like, laminin and collagen, pivotal in aggressive cancer phenotypes. **(F)** Potential ligand-receptor interactions, inferred through NicheNet analysis, emphasize activity differences between cell types across MLTS groups, identifying key interactions that may guide targeted therapies. **(G)** A Circos plot summarizes top-predicted ligand-receptor pairs, pointing to heightened interactions, especially involving HMGB1-SDC1 in high MLTS cells, indicative of aggressive behavior. **(H)** A detailed map of the routes of THBS1 ligands and HMGB1 ligands to the target receptor SDC1.

The intercellular communication of these 7 types of cells in MLTS was evaluated by Cell Chat analysis. The interaction numbers and strength were stronger in the low MLTS cells (**Figures 6A, B**). Signaling pathways analysis further showed that there were stronger intercellular communications in most of the signaling pathways in the low MLTS group (**Figure 6C**). For example, epithelial cells were alleviated form the signals of macrophages, and the gene expression of collagen was significantly different between two MLTS groups in epithelial cells (**Supplementary Figures S5A, B**). Moreover, the outgoing and incoming interaction strength was deployed to monitor the cell-cell interactions (**Figure 6D**). The results showed the changes in the intensity of outgoing and incoming signals in different cells, in which stronger incoming interactions among epithelial cells, T cells, macrophages and pericytes in low MLTS group, accompanied by stronger outgoing interactions between pericytes and fibroblasts. Various pathways in epithelial cells are specific in low MLTS group, for instance, outgoing signals were specific in both Collagen and Laminin pathways, and whether the incoming and outgoing interactions of different cells were enhanced in the specific signaling pathways was also exhibited in detail (**Figure 6E**; **Supplementary Figures S5C, D**).

The effects of different cells on TME epithelial cells were explored by nichenetr analysis, and some potential ligands that could specifically regulate epithelial cells were speculated in the two MLTS groups (**Supplementary Figure S5E**). One of the most interesting ligands based on the carcinogenic effect during development tumor was HMGB1. HMGB1 exhibited a stronger ligand activity in T cells and fibroblasts compared with macrophages and pericytes (**Supplementary Figure S5F**), and a significant difference in the amount of this ligand was observed in pericytes (**Supplementary Figure S5G**). Depper analyses revealed the differential activity of ligand-receptor pairs for MLTS, we summarize the top-predicted links in the circos plot (**Figure 6F**). A high degree of interaction between HMGB1-SDC1 was observed in cells in low MLTS group (**Figure 6G**), indicating that fibroblasts and T cells may be the main sending cells of affecting changes in the epithelial cell pathway. **Figure 6H** shows a detailed roadmap of HMGB1 ligand reaching the target receptor SDC1 through other receptors or transcription factors.

### Evaluation of potential immunotherapeutic targets of MLTS

**Figure 7:**
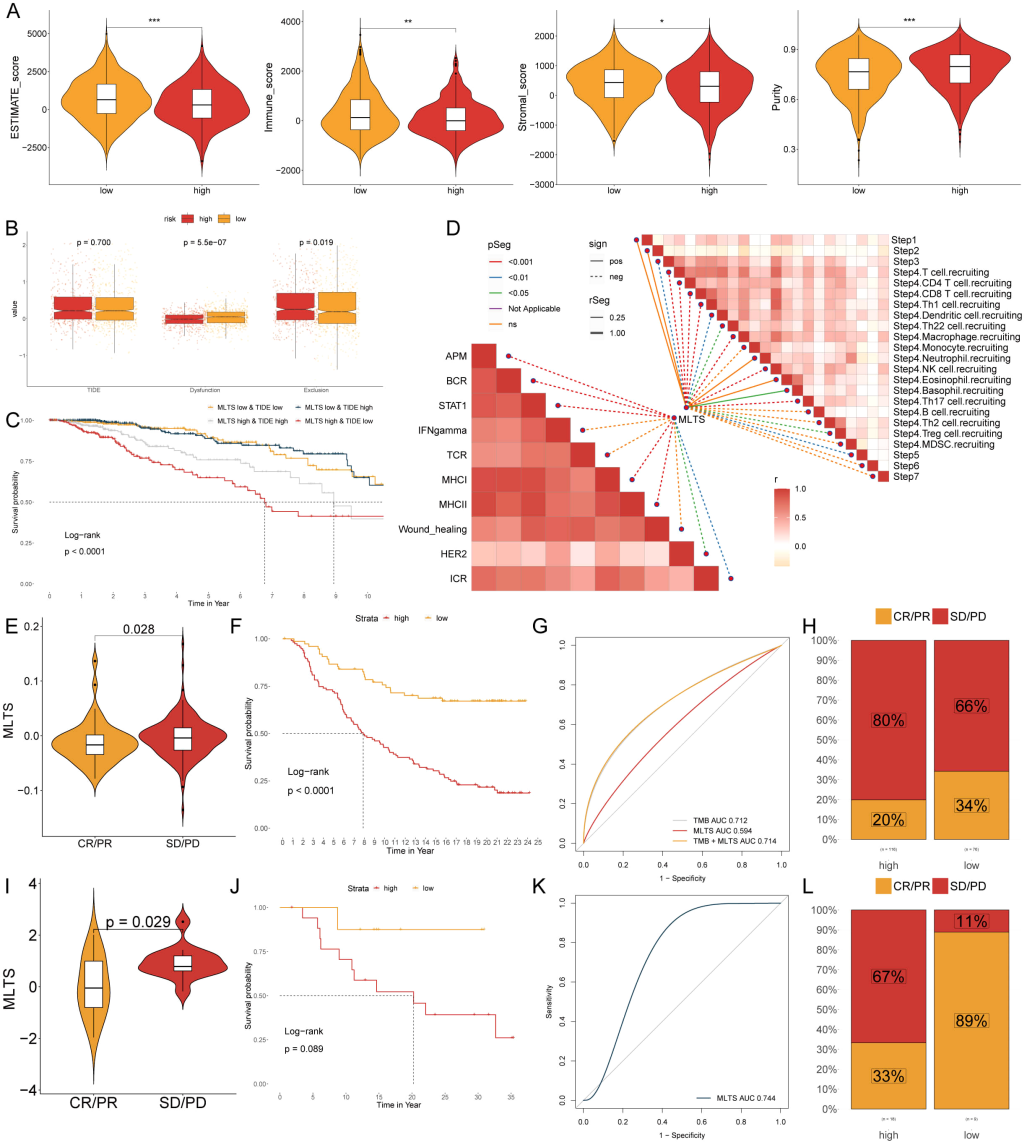
MLTS correlation with immune infiltration and response to immunotherapy in breast cancer. **(A)** Violin plots illustrate the comparison of ESTIMATE, immune, and stromal scores between low and high MLTS groups, highlighting the variation in the tumor microenvironment influenced by MLTS levels. **(B)** Box plots demonstrate TIDE values, dysfunction, and exclusion metrics for patients with low versus high MLTS, providing insights into immune evasion strategies associated with different MLTS levels. **(C)** Kaplan-Meier survival curves display the survival outcomes for breast cancer patients stratified by MLTS and TIDE scores, revealing differences in patient prognoses based on MLTS. **(D)** Correlation analysis of MLTS with different stages and signaling pathways of tumor immunity. **(E, I)** Violin charts display the relationship between MLTS levels and responses to anti-PDL1 **(E)** and anti-PD1 **(I)** therapies, detailing the differential immune responses. **(F, J)** Survival probabilities of low and high MLTS patients in anti-PDL1 **(F)** and anti-PD1 **(J)** cohorts, respectively, illustrating the impact of MLTS on survival outcomes. **(G, K)** Analysis estimates the predictive ability of MLTS via AUC values, considering TMB combinations, in anti-PDL1 **(G)** and anti-PD1 **(K)** cohorts, evaluating the efficacy of MLTS as a biomarker. **(H, L)** The percentages of complete response/partial response (CR/PR) and stable disease/progressive disease (SD/PD) in anti-PDL1 **(H)** and anti-PD1 **(L)** cohorts are shown, based on MLTS levels, to assess treatment effectiveness. *p < 0.05, **p < 0.01, ***p < 0.001.

Due to the correlation between the immune microenvironment and tumor progression, six algorithms were applied to evaluate the immune infiltration. A higher proportion of immune cell infiltration was found in the low MLTS patients, while only a few immune cell infiltrations was observed in the high MLTS patients (**Supplementary Figure S6A**). The low MLTS patients were superior in estimated scores, immune scores, and stromal scores, while the high MLTS patients showed a greater advantage in tumor purity (**Figure 7A**), indicating that the former is more likely to be inclined towards immunotherapy. It was found in the routine analysis of TIDE that Dysfunction and Exclusion values of patients were higher in the low MLTS group, but no significant difference was found in TIDE values between the MLTS groups (**Figure 7B**). To better stratify patients, patients were compared and analyzed with TIDE and MLTS (**Figure 7C**). The correlation between the steps of tumor immune cycle 1-7 and the ten major signaling pathways related to breast cancer was analyzed, and it was found that MLTS was related to most of the signaling pathways and tumor immune cycle in the breast cancer (**Figure 7D**).

The expression level of immune checkpoint inhibitors (ICIs) is considered a key indicator for evaluating the responsiveness to immunotherapy. The results showed that many immune checkpoints were elevated in the low MLTS patients, while fewer immune checkpoints were observed higher in the high MLTS group (**Supplementary Figure S6B**), suggesting that the low MLTS patients may be more suitable for immunotherapy, as there are more immune checkpoints in their bodies, so multi-target immunotherapy can be used in them. IHC was performed to support the above results using the representative cell markers and clinical ICIs (**Supplementary Figure S6D**). MLSG in the IMvigor210 (anti-PD-L1) and GSE78220 (anti-PD-1) cohorts was further evaluated. In IMvigor210 (**Figures 7E-H**) and GSE78220 (**Figures 7I-L**), patients with low MLTS had better survival rates and clinical benefits than those with high MLTS. In summary, low MLTS patients may benefit better from ICIs treatment.

### Identification of therapeutic drugs for high MLTS patients

**Figure 8:**
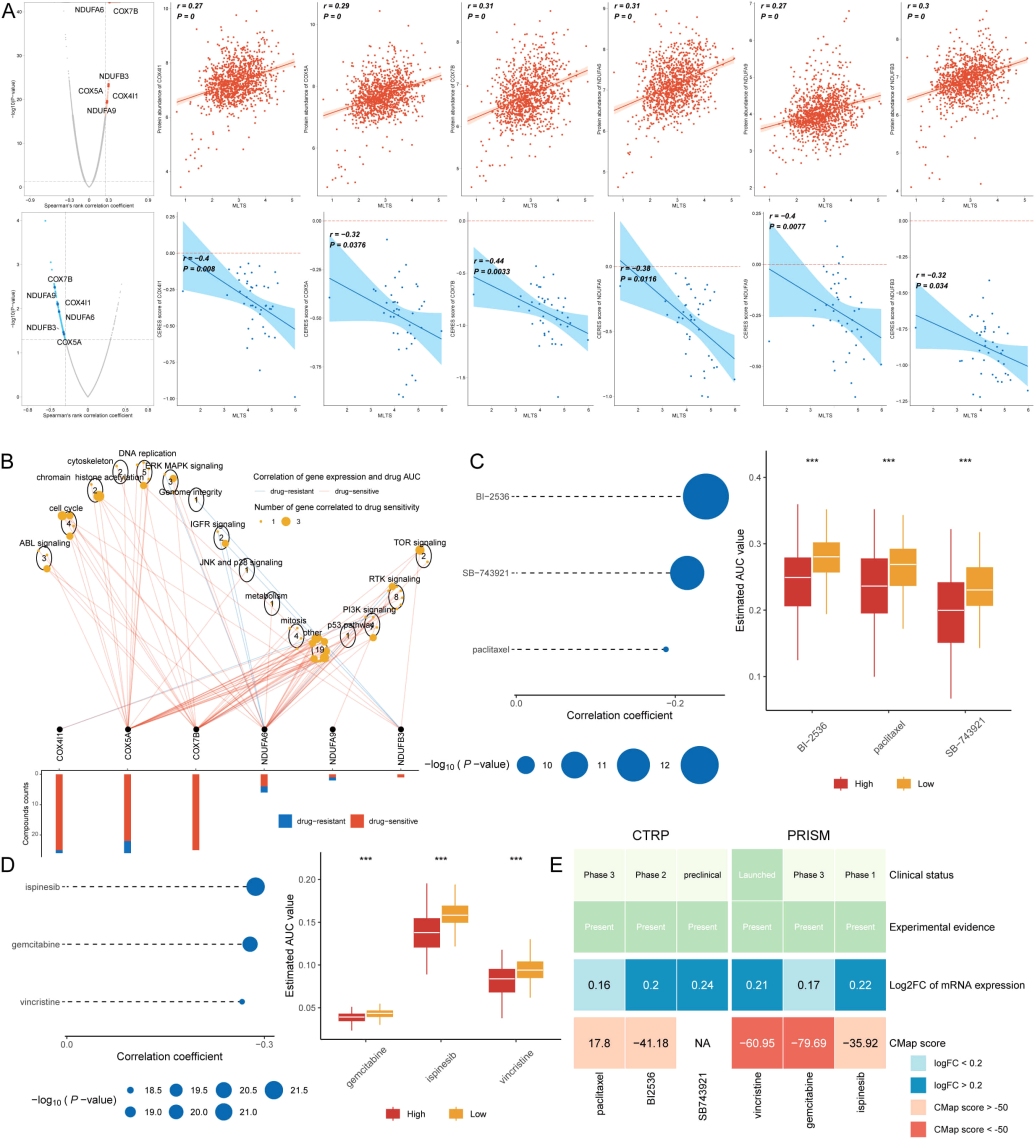
Screening therapeutic targets and drugs for high MLTS breast cancer patients. **(A)** Scatter plots display Spearman’s correlation coefficients, illustrating the association between MLTS and the abundance of six potential therapeutic targets in breast cancer patients. A notable negative correlation with CERES scores suggests these targets could be especially significant for patients with high MLTS, potentially offering new avenues for targeted therapy. **(B)** Network analysis highlights the intricate connections between these six therapeutic targets and their associated drug action pathways, indicating significant implications for the development of therapeutics for breast cancer patients with high MLTS and underscoring their potential as critical intervention points. **(C, D)** Box plots compare the AUC values of nine compounds, sourced from the CTRP and PRISM datasets, between low and high MLTS patient groups. Observations of higher AUC values in low MLTS patients indicate less favorable chemotherapy outcomes for this subgroup, pointing to the need for personalized treatment strategies. **(E)** A summary table outlines the multi-perspective analysis of the nine candidate compounds, detailing their clinical status, experimental evidence, mRNA expression levels, and CMap scores. Vincristine and gemcitabine are highlighted as a potentially suitable therapeutic agent for high MLTS patients based on its favorable CMap score, suggesting it could be particularly effective in this patient subset. ****p < 0.0001.

Chemotherapy remains a cornerstone treatment for cancer. In this study, we developed a targeted approach for breast cancer patients exhibiting high MLTS levels, utilizing sensitivity data gathered from multiple datasets. Initially, we used Spearman’s correlation analysis to identify key therapeutic targets. The analysis indicated a positive correlation between MLTS and the abundance of six potential targets (NDUFA6, COX7B, NDUFB3, COX5A, COX4I1, NDUFA9); importantly, these targets also showed notably negative correlations with their respective CERES scores, suggesting their viability as therapeutic targets for patients with high MLTS levels (**Figure 8A**). Additionally, these six targets were found to be closely linked to various drug action pathways, underscoring their significance as critical therapeutic targets for this patient subgroup (**Figure 8B**).

Subsequently, we procured nine chemical compounds from the CTPR and PRISM datasets, including SB-743921, paclitaxel, BI2536, vincristine, gemcitabine, and ispinesib. An analysis comparing the AUC values of these compounds between two MLTS groups revealed higher AUC values in patients with low MLTS, indicating a less favorable response to chemotherapy in this demographic (**Figures 8C, D**). A comprehensive multiple-perspective analysis was then conducted to select the most effective therapeutic drugs from these nine candidates. This analysis included detailed evaluations of each compound’s clinical status, experimental evidence, mRNA expression levels, and Connectivity Map (CMap) scores. Ultimately, vincristine and gemcitabine were identified as the most suitable therapeutic drug for patients with high MLTS, based on its CMap score (**Figure 8E**).

## Discussion

Brest cancer is the main malignant tumor that endangers women’s health and remains an important disease that causes female mortality despite in recent years. Currently, prognostic measures for breast cancer remain suboptimal, with a lack of widely applicable prognostic prediction models in clinical practice. Telomerase activation is detected in approximately 80%-90% of human cancer cells but is absent in normal somatic cells, suggesting that telomerase detection could enhance cancer prognostic diagnoses (5, 27). Telomerase has been extensively studied as a therapeutic target in breast cancer due to its role in maintaining genomic stability and promoting tumor cell survival. Recent studies highlight its potential as a drug target, offering new avenues for therapeutic intervention aimed at inhibiting telomerase activity in cancer cells (28). Given the potential role of telomerase genes in malignant tumor progression, this study has developed a prognostic model based on telomerase gene activity to enhance the accuracy of breast cancer prognosis predictions.

Key telomerase genes were screened using the RSF algorithm. Finally, six telomerase genes related to breast cancer prognosis were identified and used for establishing a prognostic model. The results showed that the survival rate of patients in high MLTS group was low, and the distribution of these six telomerase genes in subgroups was significantly different. The expression levels of JAK2 and DAXX genes were higher in low MLTS patients, while those of four telomerase genes, HMGB3, XRCC4, RGS3, and PFKL, were higher in high MLTS patients. Janus kinase 2 (JAK2) is an intracellular enzyme that promotes the growth, death, and differentiation of cells. Balko JM proposed that JAK2 might be a therapeutic target for triple-negative breast cancers with amplification of JAK2, and specific JAK2 inhibitors could be used in combination with chemotherapy to inhibit this process, to reduce tumor-initiating stem cell-like cells and inhibit the tumor growth. These findings are of great significance for the targeting development of selective JAK2 inhibitors in triple-negative breast cancer and basal-like breast carcinoma (BLbreast cancer) patients (29). Death domain-associated protein (DAXX) is a multifunctional protein that can interact with various cytokines, cell proteins, or viral proteins to inhibit viral transcription (30). The overexpression of DAXX is a common feature of various cancers, and is associated with tumor occurrence, disease progression (31). Hussien Marwa Tet al.’s study demonstrated that the low expression of DAXX was associated with the poor prognosis in breast cancer, indicating that the mechanism-mediated by DAXX as a target may have the potential to treat different types of cancer (32). In summary, combining the previous studies with our study can enhance the persuasiveness of JAK2 and DAXX as candidate genes for breast cancer treatment.

Human high mobility group protein B3 (HMGB3) plays a crucial role in the repair, recombination, transcription, and replication of DNA. HMGB3 is highly expressed in many malignant tumors, and it can promote the growth of tumor cells, it can also be used to predict poor prognoses. Zhou et al. found that the expression of HMGB3 was higher in breast cancer cells, a knockdown of HMGB3 can stimulate breast cancer cell proliferation and improve chemotherapy sensitivity (33). X-ray repair cross-complementing protein 4 (XRCC4) is an important part of the DNA double-strand break repair pathway NHEJ. Wen Y et al. pointed out that XRCC4 gene knockout could enhance the sensitivity of triple-negative breast cancer cells to ionizing radiation, which could be used as a new predictor of radiation sensitivity and expected to become a target of triple-negative breast cancer (34). According to Hong Z, et al., regulator of G-protein signaling 3 (RGS3) found miR-126-3 played an inhibitory role in regulating the activity of triple-negative breast cancer cells by targeting RGS3, suggest that miR-126-3p/RGS3 axis may be a potential therapeutic target for triple-negative breast cancer (35). L-phosphofructose kinase (PFKL) glycolysis is the key for the occurrence and progression of tumors, which is associated with the poor prognosis of cancers. The research results of Wang J et al. show that hypoxia-related features of PFKL gene can serve as a potential biomarker for the prognosis of breast cancer (36). HMGB3, XRCC4, RGS3 and PFKL have been identified as potential target genes for breast cancer in many studies, providing more directions for the treatment of breast cancer patients.

Our genome-level analysis revealed a high mutation rate of the tumor suppressor gene TP53 in high MLTS patients, which appears to play a significant role in driving poor prognosis. TP53, often referred to as the “guardian of the genome,” is crucial for maintaining genomic stability by regulating cell cycle arrest, DNA repair, and apoptosis in response to cellular stress. Mutations in TP53 disrupt these tumor-suppressive functions, leading to uncontrolled cell proliferation, evasion of apoptosis, and increased susceptibility to additional genetic alterations, thereby fostering an environment conducive to tumor progression and metastasis (37). KOçAK et al. found that the prognosis of diagnosed breast cancer was poor in young women with a high copy number alteration and TP53 mutation level (38), have significantly worse clinical outcomes, further supporting the role of these mutations in poor prognosis. In our study, high MLTS patients showed not only an increased frequency of TP53 mutations but also significant amplifications in oncogenes such as those located at 5p15.33 and deletions in tumor suppressors like CDKN2A and loci on 9p23. These alterations may lead to chromosomal instability, creating a permissive environment for tumor heterogeneity and further oncogenic mutations, ultimately exacerbating the tumor’s aggressiveness and its resistance to standard therapies. Some gene expression levels on chromosomes also changed, and the amplification and deletion of chromosome arms in the high MLTS group were more pronounced, which may disrupt the normal functional order of genomic genes, and the poor prognosis of high MLTS patients may be related to the significant increase of multiple oncogenes such as 5p15.33 and the deletion of multiple tumor suppressor genes such as 9p23. Wilhelm et al.’s study showed that the chromosomal instability is a marker associated with the poor prognosis of cancers (39), so it is speculated that high-level mutations in tumor suppressor genes and chromosomal copy number alterations may be associated with the poor prognosis of breast cancer, and the poor prognosis of breast cancer is positively correlated with the frequency of these mutations. Together, these findings emphasize the critical role of TP53 mutations and chromosomal alterations in shaping the tumor microenvironment, influencing breast cancer progression, and determining patient outcomes. Understanding these molecular mechanisms not only strengthens the biological relevance of our findings but also underscores the importance of developing novel therapeutic strategies that specifically target these genetic vulnerabilities to improve prognosis for high MLTS breast cancer patients.

According to the correlation between telomerase gene expression and immune infiltration, it has been found that there is a higher proportion of CD4^+^ T cells, CD8^+^ T cells and macrophage infiltration in breast cancer patients. Some studies suggest that the poor prognosis of breast cancer is related to the infiltration of highly immunosuppressive T cells and macrophages within the tumor (40, 41). Tumor-specific CD4^+^ T cells play a crucial role in the immune response against cancers, promoting the activation of cytotoxic CD8^+^ T cells to increase their ability to destroy tumors (42, 43). Therefore, it is speculated that high levels of CD4^+^ T cell infiltration and CD8^+^ T cell infiltration are related to the poor prognosis of breast cancer. CD8^+^ T cells are crucial effector cells in the anti-tumor immune response, capable of recognizing and directly killing tumor cells. In our analysis, the infiltration level of CD8^+^ T cells was significantly higher in the low MLTS group compared to the high MLTS group, correlating with better responsiveness to immunotherapy. This suggests that the tumor microenvironment in low MLTS patients is more favorable for activating CD8^+^ T cells, enhancing their ability to target and eliminate cancer cells (42, 43). Macrophages have been proved to be associated with the poor prognosis in many cancers (44, 45). Macrophages in the TME play a complex dual role, categorized into M1 (pro-inflammatory) and M2 (immunosuppressive) phenotypes. M2 macrophages are typically associated with tumor growth, metastasis, and immune evasion. In the high MLTS group, we observed a significant increase in M2 macrophages, which might contribute to poor immunotherapy responsiveness and worse prognosis in these patients (44, 45). The results of the analysis on the cell-to-cell communication, the intercellular interaction network and the intensity of incoming and outgoing interactions showed that the number and intensity of communication between macrophages and fibroblasts in breast cancer tissue increased significantly, also indicating that macrophages may be related to the poor prognosis of breast cancer.

Based on the immune cell infiltration scores and the expression of immune checkpoints in the two MLTS subgroups, it was found that the immune cell infiltration in patients in the low MLTS group was more significant, and the expression of immune checkpoints in the low MLTS group was much higher than that in the high MLTS group. ŚLEDZIŃSKA et al. reported that immune checkpoints play a dominant role in malignant tumors and can prevent the effective anti-tumor immune response. However, manipulating these immune checkpoints with therapeutic antibodies can control or even eliminate tumors, which may become a new method for tumor immunotherapy (46). It can be inferred that patients in the low MLTS group are in an immune activated state, while those in the high MLTS group are in an immunosuppressive state. To better compare the two groups and determine which would be more suitable for immunotherapy, multiple methods were used in this study. Our analysis showed significantly higher expression levels of immune checkpoint molecules in the low MLTS group, indicating that these patients might be in an immune-activated state and more suitable for immune checkpoint inhibitor therapy. In contrast, the high MLTS group had lower expression of immune checkpoints, suggesting an immunosuppressive state that might make these patients less responsive to immunotherapy (46). The results showed that the low MLTS patients were more suitable for immunotherapy than the high MLTS patients, and low MLTS patients could benefit from ICIs. Chemotherapy is currently one of the standard therapies for cancers. Six specific targets associated with MLTS (NDUFA6, COX7B, NDUFB3, COX5A, COX411, and NDUFA9) were screened out, so it is speculated that these six targets may serve as potential therapeutic targets for high MLTS patients. Finally, six compounds related to MLTS were identified from two datasets, and the comparison on AUC values in the two MLTS subgroups showed that patients in high MLTS group were more suitable for chemotherapy, however, gemcitabine and vincristine were ultimately identified as potential therapeutic drugs for high MLTS breast cancer patients.

This study’s findings not only illuminate the pivotal role of telomerase gene expressions in breast cancer progression but also underscore the transformative potential of machine learning in medical prognostics. Our analysis, built on a novel predictive model, reveals a significant correlation between telomerase activity and patient outcomes, highlighting its utility as a prognostic biomarker. Furthermore, the integration of machine learning has allowed for a nuanced interpretation of complex genetic data, leading to more precise predictions than those offered by traditional methods. This approach has shown its capacity to handle the intricate patterns of gene expressions, providing a robust framework for future research aiming to expand upon these findings.

While our study presents a comprehensive approach to understanding breast cancer prognosis using the MLTS, there are several limitations that should be acknowledged. First, the data used for model development and validation were primarily derived from publicly available datasets such as TCGA and GEO. Although these datasets provide valuable resources for large-scale analysis, they may introduce biases due to the lack of representation of diverse patient populations, ethnic groups, and clinical subtypes. As a result, the generalizability of our findings might be limited when applied to populations not included in these datasets. Future studies should aim to validate our model in more geographically and ethnically diverse cohorts to assess its robustness across different clinical settings. The use of machine learning models in this study, while offering significant predictive power, also comes with inherent challenges. One limitation is the risk of overfitting, particularly when using complex models like Random Forest, Gradient Boosting Machine, and Survival-SVM. Overfitting can occur when the model learns noise or specific patterns in the training data that do not generalize well to new, unseen data. To mitigate this issue, we employed cross-validation and parameter optimization techniques, but the potential for overfitting remains a concern. Additionally, machine learning models often operate as “black boxes,” where the interpretation of results can be difficult, especially in understanding the contribution of individual features to the model’s predictions. Further work is needed to explore interpretable machine learning techniques that can provide more insight into the decision-making process of these models.

Interpreting scRNA-seq data poses several challenges that can affect the reliability of our findings. One major issue is technical variability, which includes factors like sequencing depth, batch effects, and data dropout, where genes may appear as unexpressed due to insufficient sensitivity in detection. These factors can lead to inconsistencies in gene expression profiles and impact the identification of cell types or states. While we applied rigorous data preprocessing and quality control measures, such as filtering cells with high mitochondrial gene content and using normalization techniques, these challenges highlight the need for careful interpretation of scRNA-seq results. Moreover, the inherent sparsity and noise in scRNA-seq data can complicate downstream analyses, such as clustering and trajectory inference, potentially affecting the robustness of the conclusions drawn from these datasets.

Our study highlights the robust predictive power of the MLTS in forecasting breast cancer prognosis, but its true potential lies in its application within clinical settings. Integrating MLTS into routine clinical practice could significantly enhance personalized treatment strategies by enabling clinicians to stratify patients based on their risk profiles. High-risk patients identified through MLTS could benefit from more aggressive treatment regimens or closer monitoring, while low-risk patients might be spared from unnecessary toxic therapies, thereby improving their quality of life.

## Conclusion

The implications of these findings are profound, suggesting that incorporating machine learning into cancer research can greatly enhance the precision of prognostic models. This advancement holds promise for personalized medicine, where tailored therapies can be developed based on individual genetic profiles, potentially leading to better patient outcomes. It appears that patients in the low MLTS group may respond better to immunotherapy, while those in the high MLTS group may respond better to chemotherapy. The screened six potential targets for breast cancer patients in high MLTS group and two potential therapeutic drugs found in this study may provide a new treatment option for high MLTS breast cancer patients.

## Data Availability

The original contributions presented in the study are included in the article/**Supplementary Material**. Further inquiries can be directed to the corresponding author.

## References

[1] Bray F Ferlay J Soerjomataram I Siegel RL Torre LA Jemal A. Global cancer statistics 2018: GLOBOCAN estimates of incidence and mortality worldwide for 36 cancers in 185 countries. CA: A Cancer J Clin. (2018) 68(6):394–424. doi: 10.3322/caac.21492 30207593

[2] Sui S An X Xu C Li Z Hua Y Huang G. An immune cell infiltration-based immune score model predicts prognosis and chemotherapy effects in breast cancer. Theranostics. (2020) 10:11938–49. doi: 10.7150/thno.49451 PMC7667685 33204321

[3] van Veen EM Brentnall AR Byers H Harkness EF Astley SM Sampson S. Use of single-nucleotide polymorphisms and mammographic density plus classic risk factors for breast cancer risk prediction. JAMA Oncol. (2018) 4:476–82. doi: 10.1001/jamaoncol.2017.4881 PMC5885189 29346471

[4] Shay JW Wright WE. Telomeres and telomerase: three decades of progress. Nat Rev Genet. (2019) 20:299–309. doi: 10.1038/s41576-019-0099-1 30760854

[5] Dan J Zhou Z Wang F Wang H Guo R Keefe DL. Zscan4 contributes to telomere maintenance in telomerase-deficient late generation mouse ESCs and human ALT cancer cells. Cells. (2022) 11(3):456. doi: 10.3390/cells11030456 35159266 PMC8834411

[6] Braun DM Chung I Kepper N Deeg KI Rippe K. TelNet - a database for human and yeast genes involved in telomere maintenance. BMC Genet. (2018) 19:32. doi: 10.1186/s12863-018-0617-8 29776332 PMC5960154

[7] Liu Z Guo C Dang Q Wang L Liu L Weng S. Integrative analysis from multi-center studies identities a consensus machine learning-derived lncRNA signature for stage II/III colorectal cancer. EBioMedicine. (2022) 75:103750. doi: 10.1016/j.ebiom.2021.103750 34922323 PMC8686027

[8] Wang L Liu Z Liang R Wang W Zhu R Li J. Comprehensive machine-learning survival framework develops a consensus model in large-scale multicenter cohorts for pancreatic cancer. Elife. (2022) 25(11):e80150. doi: 10.7554/eLife.80150 PMC9596158 36282174

[9] Pal B Chen Y Vaillant F Capaldo BD Joyce R Song X. A single-cell RNA expression atlas of normal, preneoplastic and tumorigenic states in the human breast. EMBO J. (2021) 40:e107333. doi: 10.15252/embj.2020107333 33950524 PMC8167363

[10] McGinnis CS Murrow LM Gartner ZJ. DoubletFinder: doublet detection in single-cell RNA sequencing data using artificial nearest neighbors. Cell Syst. (2019) 8:329–337.e4. doi: 10.1016/j.cels.2019.03.003 30954475 PMC6853612

[11] Suo S Zhu Q Saadatpour A Fei L Guo G Yuan GC. Revealing the critical regulators of cell identity in the mouse cell atlas. Cell Rep. (2018) 25:1436–1445.e3. doi: 10.1016/j.celrep.2018.10.045 30404000 PMC6281296

[12] Baran Y Bercovich A Sebe-Pedros A Lubling Y Giladi A Chomsky E. MetaCell: analysis of single-cell RNA-seq data using K-nn graph partitions. Genome Biol. (2019) 20:206. doi: 10.1186/s13059-019-1812-2 31604482 PMC6790056

[13] Jin S Guerrero-Juarez CF Zhang L Chang I Ramos R Kuan CH. Inference and analysis of cell-cell communication using CellChat. Nat Commun. (2021) 12:1088. doi: 10.1038/s41467-021-21246-9 33597522 PMC7889871

[14] Browaeys R Saelens W Saeys Y. NicheNet: modeling intercellular communication by linking ligands to target genes. Nat Methods. (2020) 17:159–62. doi: 10.1038/s41592-019-0667-5 31819264

[15] Becht E Giraldo NA Lacroix L Buttard B Elarouci N Petitprez F. Estimating the population abundance of tissue-infiltrating immune and stromal cell populations using gene expression. Genome Biol. (2016) 17:218. doi: 10.1186/s13059-016-1070-5 27765066 PMC5073889

[16] Racle J Gfeller D. EPIC: A tool to estimate the proportions of different cell types from bulk gene expression data. Methods Mol Biol (Clifton N.J.). (2020) 2120:233–48. doi: 10.1007/978-1-0716-0327-7_17 32124324

[17] Aran D Hu Z Butte AJ. xCell: digitally portraying the tissue cellular heterogeneity landscape. Genome Biol. (2017) 18:220. doi: 10.1186/s13059-017-1349-1 29141660 PMC5688663

[18] Newman AM Liu CL Green MR Gentles AJ Feng W Xu Y. Robust enumeration of cell subsets from tissue expression profiles. Nat Methods. (2015) 12:453–7. doi: 10.1038/nmeth.3337 PMC4739640 25822800

[19] Finotello F Mayer C Plattner C Laschober G Rieder D Hackl H. Molecular and pharmacological modulators of the tumor immune contexture revealed by deconvolution of RNA-seq data. Genome Med. (2019) 11:34. doi: 10.1186/s13073-019-0638-6 31126321 PMC6534875

[20] Li T Fan J Wang B Traugh N Chen Q Liu JS. TIMER: A web server for comprehensive analysis of tumor-infiltrating immune cells. Cancer Res. (2017) 77:e108–10. doi: 10.1158/0008-5472.Can-17-0307 PMC6042652 29092952

[21] Jiang P Gu S Pan D Fu J Sahu A Hu X. Signatures of T cell dysfunction and exclusion predict cancer immunotherapy response. Nat Med. (2018) 24:1550–8. doi: 10.1038/s41591-018-0136-1 PMC6487502 30127393

[22] Yoshihara K Shahmoradgoli M Martinez E Vegesna R Kim H Torres-Garcia W. Inferring tumour purity and stromal and immune cell admixture from expression data. Nat Commun. (2013) 4:2612. doi: 10.1038/ncomms3612 24113773 PMC3826632

[23] Meyers RM Bryan JG McFarland JM Weir BA Sizemore AE Xu H. Computational correction of copy number effect improves specificity of CRISPR-Cas9 essentiality screens in cancer cells. Nat Genet. (2017) 49:1779–84. doi: 10.1038/ng.3984 PMC5709193 29083409

[24] Yang C Huang X Li Y Chen J Lv Y Dai S. Prognosis and personalized treatment prediction in TP53-mutant hepatocellular carcinoma: an in silico strategy towards precision oncology. Brief Bioinform. (2021) 22(3):bbaa164. doi: 10.1093/bib/bbaa164 32789496

[25] Wang T Li T Li B Zhao J Li Z Sun M. Immunogenomic landscape in breast cancer reveals immunotherapeutically relevant gene signatures. Front Immunol. (2022) 13:805184. doi: 10.3389/fimmu.2022.805184 35154121 PMC8829007

[26] Wang T Ba X Zhang X Zhang N Wang G Bai B. Nuclear import of PTPN18 inhibits breast cancer metastasis mediated by MVP and importin β2. Cell Death Dis. (2022) 13:720. doi: 10.1038/s41419-022-05167-z 35982039 PMC9388692

[27] Harn H-J Lin S-Z Lin P-C Liu C-Y Liu P-y Chang L-F. Local interstitial delivery of z-butylidenephthalide by polymer wafers against Malignant human gliomas. Neuro-oncology. (2011) 13 6:635–48. doi: 10.1093/neuonc/nor021 PMC3107093 21565841

[28] Jaiswal RK Yadava PK. Assessment of telomerase as drug target in breast cancer. J Biosci. (2020) 45:72. doi: 10.1007/s12038-020-00045-2 32515354

[29] Balko JM Schwarz L Luo N Estrada MV Giltnane JM Davila-Gonzalez D. Triple-negative breast cancers with amplification of JAK2 at the 9p24 locus demonstrate JAK2-specific dependence. Sci Trans Med. (2016) 8:334ra53–334ra53. doi: 10.1126/scitranslmed.aad3001 PMC5256931 27075627

[30] Huang Z. Research status of death domain-associated protein. Highlights Science Eng Technol. (2022). doi: 10.54097/hset.v14i.1601

[31] Mahmud I Liao D. DAXX in cancer: phenomena, processes, mechanisms and regulation. Nucleic Acids Res. (2019) 47:7734–52. doi: 10.1093/nar/gkz634 PMC6735914 31350900

[32] Hussien MT Shaban S Temerik DF Helal SR Mosad E Elgammal S. Impact of DAXX and ATRX expression on telomere length and prognosis of breast cancer patients. J Egypt Natl Canc Inst. (2020) 32(1):34. doi: 10.1186/s43046-020-00045-1 32856116 PMC13317084

[33] Zhou X Zhang Q Liang G Liang X Luo B. Overexpression of HMGB3 and its prognostic value in breast cancer. Front Oncol. (2022) pp:1048921. doi: 10.3389/fonc.2022.1048921 PMC9815698 36620553

[34] Wen Y Dai G Wang L Fu K Zuo S. Silencing of XRCC4 increases radiosensitivity of triple-negative breast cancer cells. Bioscience Rep. (2019) 39(3):BSR20180893. doi: 10.1042/BSR20180893 PMC6423307 30842344

[35] Hong Z Hong C Ma B Wang Q Zhang X Li L. MicroRNA−126−3p inhibits the proliferation, migration, invasion, and angiogenesis of triple−negative breast cancer cells by targeting RGS3. Oncol Rep. (2019) 42:1569–79. doi: 10.3892/or.2019.7251 31364749

[36] Wang J Wang Y Xing P Liu Q Zhang C Sui Y. Development and validation of a hypoxia-related prognostic signature for breast cancer. Oncol Lett. (2020) 20:1906–1914. doi: 10.3892/ol.2020.11733 32724434 PMC7377061

[37] Vogelstein B Lane D Levine AJ. Surfing the p53 network. Nature. (2000) 408:307–10. doi: 10.1038/35042675 11099028

[38] Koçak A Heselmeyer-Haddad K Lischka A Hirsch D Fiedler D Hu Y. High levels of chromosomal copy number alterations and TP53 mutations correlate with poor outcome in younger breast cancer patients. Am J Pathol. (2020) 190:1643–56. doi: 10.1016/j.ajpath.2020.04.015 PMC7397461 32416097

[39] Wilhelm T Olziersky A-M Harry D de Sousa F Vassal H Eskat A. Mild replication stress causes chromosome mis-segregation via premature centriole disengagement. Nat Commun. (2019) 10(1):3585. doi: 10.1038/s41467-019-11584-0 31395887 PMC6687892

[40] Kos K Salvagno C Wellenstein MD Aslam MA Meijer DA Hau C-S. Tumor-associated macrophages promote intratumoral conversion of conventional CD4+ T cells into regulatory T cells via PD-1 signalling. Oncoimmunology. (2022) 11(1):2063225. doi: 10.1080/2162402X.2022.2063225 35481289 PMC9037432

[41] Osipov A Saung MT Zheng L Murphy AG. Small molecule immunomodulation: the tumor microenvironment and overcoming immune escape. J Immunotherapy Cancer. (2019) 7(1):224. doi: 10.1186/s40425-019-0667-0 PMC6704558 31439034

[42] Dudley ME Wunderlich JR Robbins PF Yang JC Hwu P Schwartzentruber DJ. Cancer regression and autoimmunity in patients after clonal repopulation with antitumor lymphocytes. Science. (2002) 298:850–4. doi: 10.1126/science.1076514 PMC1764179 12242449

[43] Peterson AC Harlin H Gajewski TF. Immunization with Melan-A peptide-pulsed peripheral blood mononuclear cells plus recombinant human interleukin-12 induces clinical activity and T-cell responses in advanced melanoma. J Clin Oncol. (2003) 21:2342–8. doi: 10.1200/jco.2003.12.144 12805336

[44] Cassetta L Pollard JW. Targeting macrophages: therapeutic approaches in cancer. Nat Rev Drug Discovery. (2018) 17:887–904. doi: 10.1038/nrd.2018.169 30361552

[45] Sylvestre M Crane CA Pun SH. Progress on modulating tumor-associated macrophages with biomaterials. Advanced Materials. (2019) 32:e1902007. doi: 10.1002/adma.201902007 31559665 PMC7098849

[46] Śledzińska A Menger L Bergerhoff KF Peggs KS Quezada SA. Negative immune checkpoints on T lymphocytes and their relevance to cancer immunotherapy. Mol Oncol. (2015) 9:1936–65. doi: 10.1016/j.molonc.2015.10.008 PMC5528732 26578451

