# Supplementary material for: Evaluating the prognostic potential of telomerase signature in breast cancer through advanced machine learning model
Source: Front Immunol. 2024 Nov 28;15:1462953. doi: 10.3389/fimmu.2024.1462953 (PMC11634871; doi:10.3389/fimmu.2024.1462953)
Supplement: Supplementary file 1 [file DataSheet1.pdf]

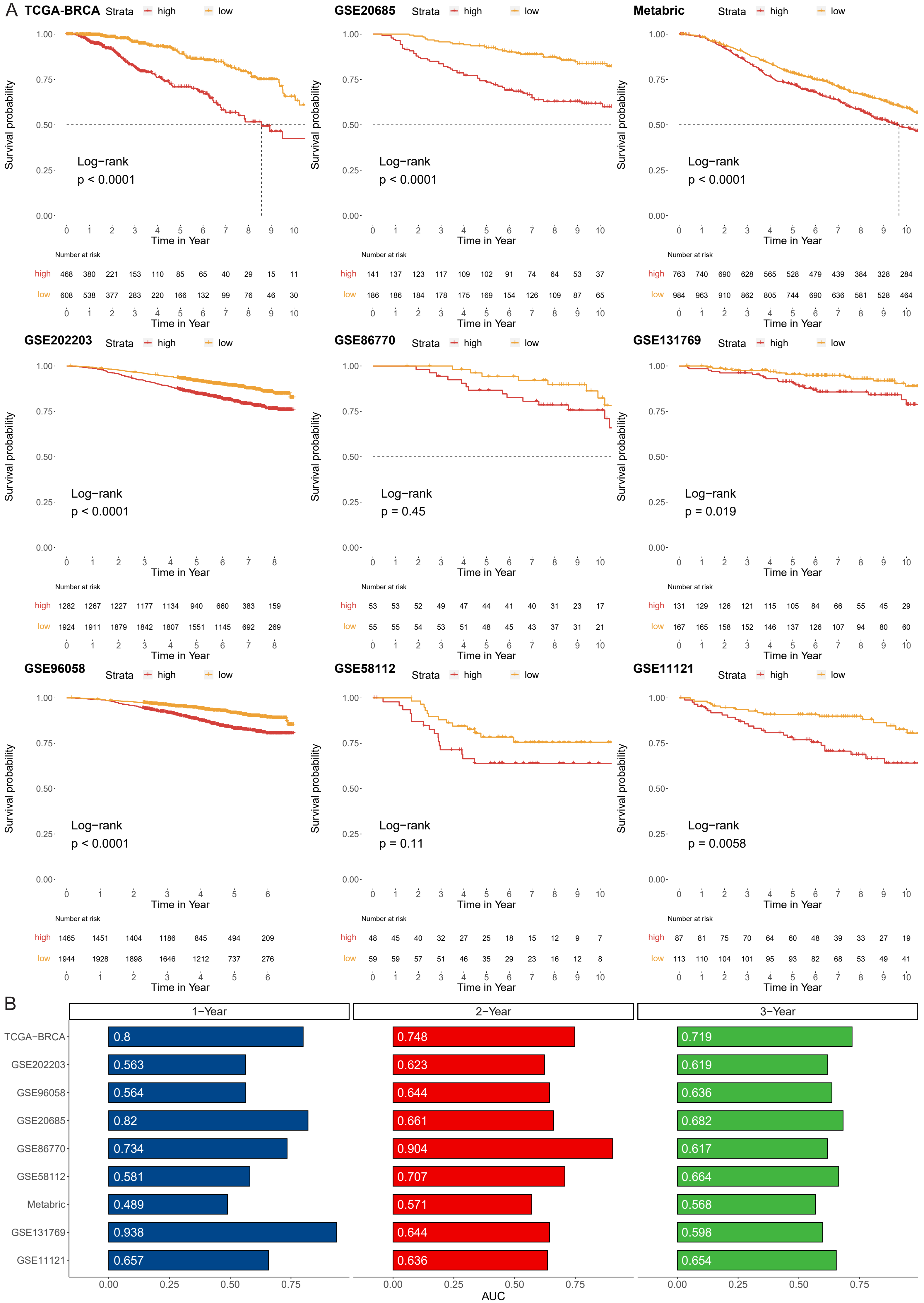

Figure S1. Evaluation of MLTS in 9 cohorts. (A) Kaplan-Meier curves of the MLTS in 9 cohorts. (B) Time-dependent ROC analysis for predicting OS at 1, 3, and 5 years.
