# Supplementary material for: Evaluating the prognostic potential of telomerase signature in breast cancer through advanced machine learning model
Source: Front Immunol. 2024 Nov 28;15:1462953. doi: 10.3389/fimmu.2024.1462953 (PMC11634871; doi:10.3389/fimmu.2024.1462953)
Supplement: Supplementary file 4 [file DataSheet4.pdf]

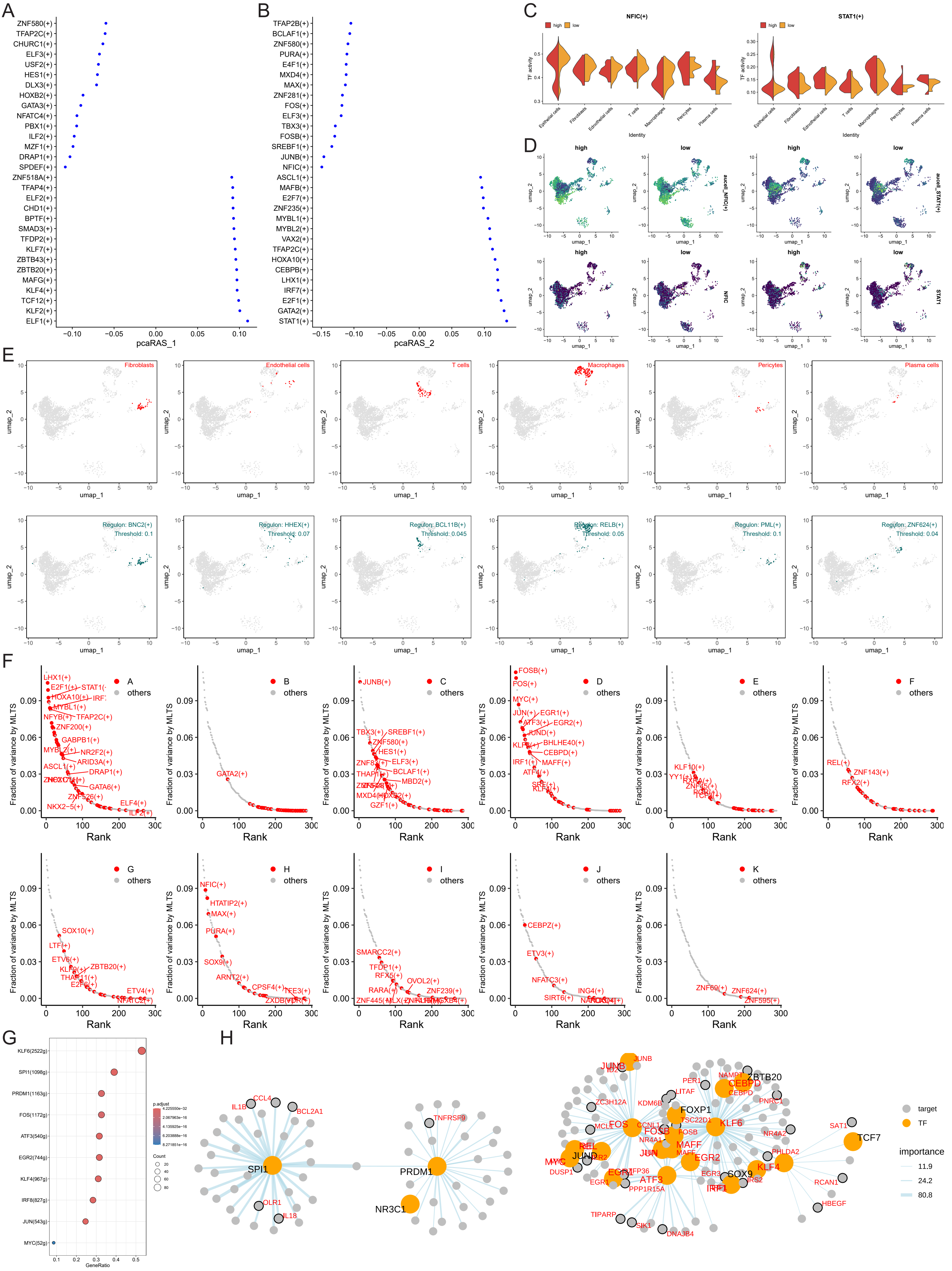

Figure S4. Transcription factor activity correlation and contribution analysis in cell types. (A) Correlation of transcription factor activity with the principal component regulatory activity score 1 (pcaRAS1), highlighting which factors exert positive or negative influences on the regulatory landscape, providing insights into their potential regulatory roles within the cellular context. (B) Correlation analysis with pcaRAS2, offering a contrasting perspective on the influence of transcription factors and further elucidating their complex roles in cellular regulation. (C) Violin plots show the distribution of transcription factor activity levels across various conditions or cell types, indicating the variability and dynamic range of transcription factor expressions. (D) Distribution maps of samples based on transcription factor activity and gene expression levels. (E) UMAP projections categorize samples by transcription factor activity, revealing distinct cell types based on their transcriptional profiles, aiding in the identification of unique cellular behaviors and properties. (F) Illustrates the contribution of different transcription factor groups to MLTS, with significant transcription factors highlighted and ranked based on their RSS, showcasing their regulatory impact on gene expression particularly in epithelial cells. (G) Identifies transcription factors that contribute to TNFA signaling via the NFKB, highlighting their critical roles in the progression of MLTS and providing a targeted view of transcriptional regulation linked to specific signaling pathways. (H) A detailed regulatory network illustrates the interactions of transcription factors involved in the TNFA signaling of MLTS progression, mapping the complex interplay and influence of these factors on the disease progression pathway.
