# Supplementary material for: Evaluating the prognostic potential of telomerase signature in breast cancer through advanced machine learning model
Source: Front Immunol. 2024 Nov 28;15:1462953. doi: 10.3389/fimmu.2024.1462953 (PMC11634871; doi:10.3389/fimmu.2024.1462953)
Supplement: Supplementary file 5 [file DataSheet5.pdf]

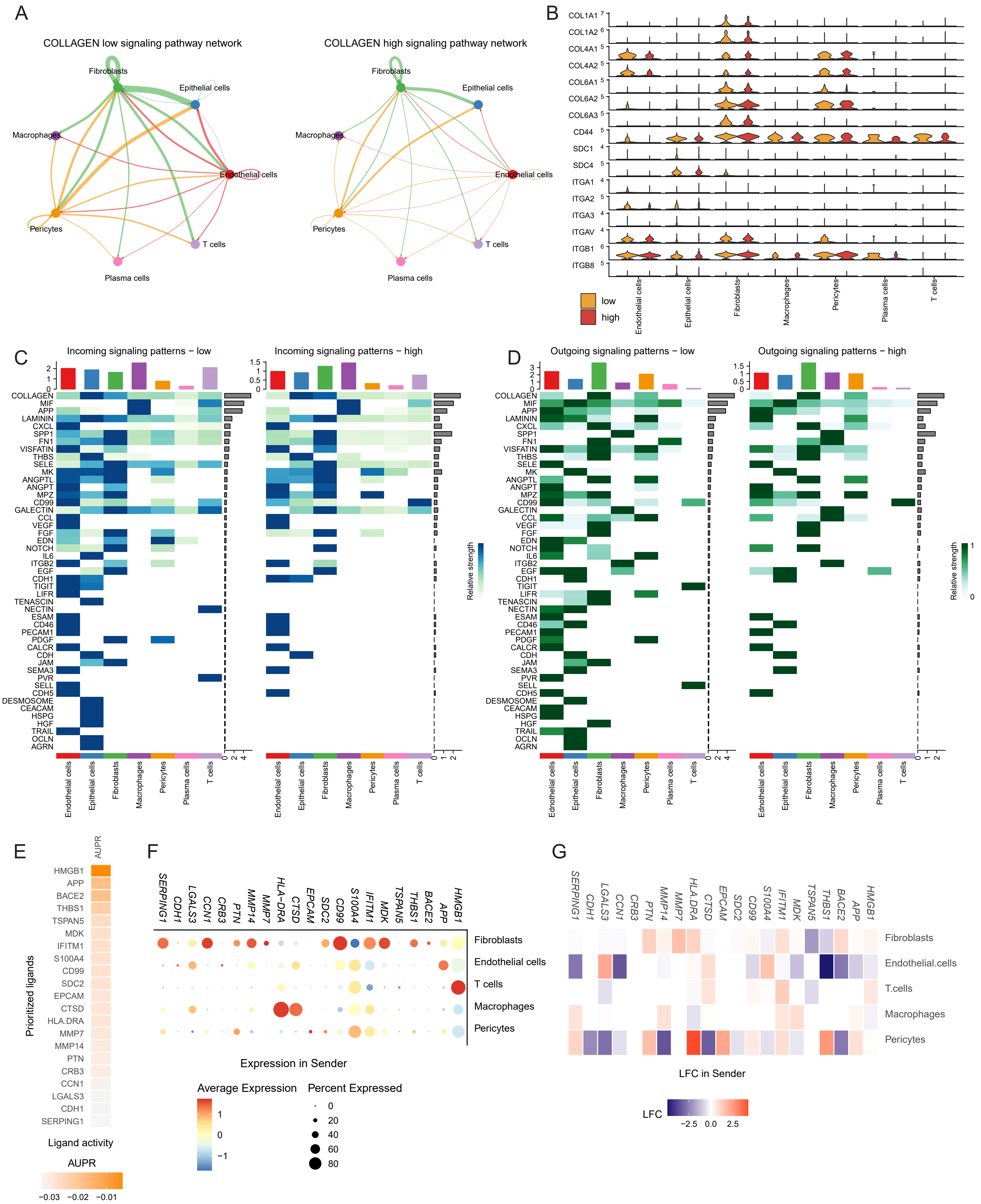

Figure S5. Cellular interactions in tumor microenvironments based on MLTS. (A) Heatmaps display the activity level of collagen signaling within various cell types in low and high MLTS groups. (B) Violin plots illustrate the expression levels of collagen and related genes across different cell populations segmented into low and high MLTS. (C) Heatmaps compare incoming signaling patterns between cell types with low and high MLTS. (D) Heatmaps show outgoing signaling patterns in cell populations with low versus high MLTS. (E) Ranks for ligand activity, categorizing the importance and impact of various ligands within the tumor microenvironment, which helps in pinpointing key signaling molecules in MLTS-dependent contexts. (F) Dot plot highlights gene expression in the sender cell types for various ligand-receptor pairs, colored and sized by expression level, which could elucidate how signaling capabilities vary among sender cells, affecting their influence on the microenvironment. (G) Log fold change (LFC) in receiver cell types provides insight into the potential responsiveness of these cells to signals, based on their gene expression profiles, indicating how different cells might react to intercellular communication.
