# Supplementary material for: Evaluating the prognostic potential of telomerase signature in breast cancer through advanced machine learning model
Source: Front Immunol. 2024 Nov 28;15:1462953. doi: 10.3389/fimmu.2024.1462953 (PMC11634871; doi:10.3389/fimmu.2024.1462953)
Supplement: Supplementary file 6 [file DataSheet6.pdf]

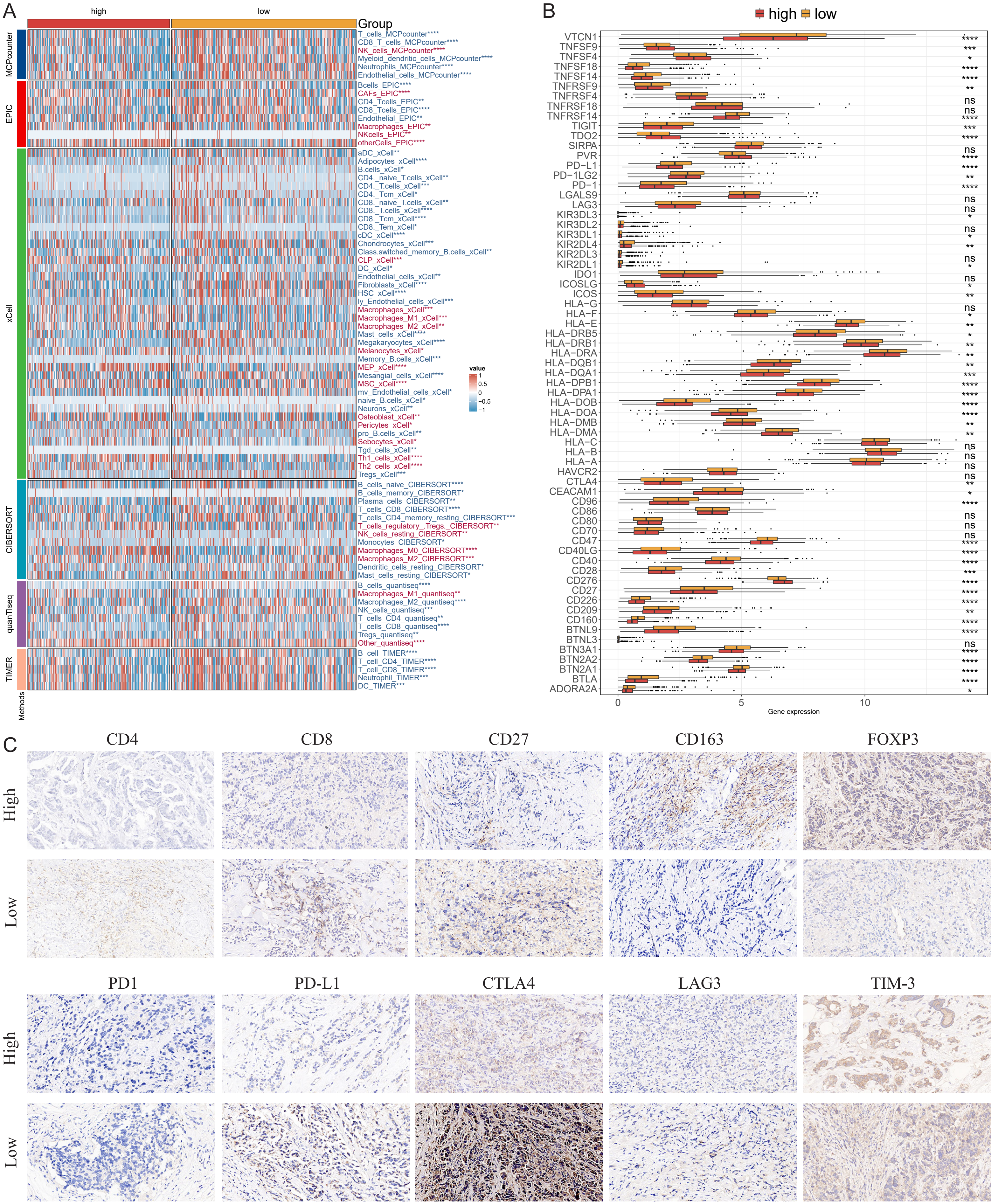

Figure S6. Differential expression and immunohistochemical analysis of immune markers in tumor microenvironments between MLTS subgroups. (A) Heatmap provides a comparative view of immune cell infiltration in tumor samples with low and high MLT, utilizing various computational algorithms for quantification. Each row represents a different type of immune cell, with the color intensity reflecting the level of infiltration. (B) Box plots illustrate the distribution of gene expression levels for immune checkpoint inhibitors (ICIs) across low vs. high MLTS conditions, with statistical significance denoted by ns for not significant; *P < 0.05; **P < 0.01; ***P < 0.001; ****P < 0.0001. (C) Representative immunohistochemistry images showcase the staining intensity of various immune markers between high and low expression conditions, visually depicting the differential expression of these markers in correlation with MLTS levels.
